# Age-dependent differences in breast tumor microenvironment: challenges and opportunities for efficacy studies in preclinical models

**DOI:** 10.1038/s41418-025-01447-1

**Published:** 2025-01-27

**Authors:** Paolo Falvo, Stephan Gruener, Stefania Orecchioni, Federica Pisati, Giovanna Talarico, Giulia Mitola, Davide Lombardi, Giulia Bravetti, Juliane Winkler, Iros Barozzi, Francesco Bertolini

**Affiliations:** 1 Center for Cancer Research, Medical University of Vienna, Borschkegasse 8A, 1090 Vienna, Austria; 2 Laboratory of Hematology-Oncology, European Institute of Oncology IRCCS, Via Ripamonti 435, 20141 Milan, Italy; 3 Department of Experimental Oncology, European Institute of Oncology IRCCS European Institute of Oncology, Via Adamello 16, 20141 Milan, Italy; 4 Onco-Tech Lab, European Institute of Oncology IRCCS and Politecnico di Milano, Milan, Italy; 5 Histopathology Unit, Cogentech Societa’ Benefit srl, Milan, Italy; 6 Present Address: ASST Brianza, Ospedale di Vimercate, Microbiologia e Virologia, Via Santi Cosma e Damiano 10, 20871 Vimercate, Italy; 7 Present Address: Data Collection G-STeP Research Core Facility, Fondazione Policlinico Universitario A. Gemelli IRCCS, 00168 Roma, Italy

**Keywords:** Cancer models, Translational research

## Abstract

Immunity suffers a function deficit during aging, and the incidence of cancer is increased in the elderly. However, most cancer models employ young mice, which are poorly representative of adult cancer patients. We have previously reported that Triple-Therapy (TT), involving antigen-presenting-cell activation by vinorelbine and generation of TCF1^+^-stem-cell-like T cells (scTs) by cyclophosphamide significantly improved anti-PD-1 efficacy in anti-PD1-resistant models like Triple-Negative Breast Cancer (TNBC) and Non-Hodgkin’s Lymphoma (NHL), due to T-cell-mediated tumor killing. Here, we describe the effect of TT on TNBC growth and on tumor-microenvironment (TME) of young (6–8w, representative of human puberty) versus adult (12 m, representative of 40y-humans) mice. TT-efficacy was similar in young and adults, as CD8^+^ scTs were only marginally reduced in adults. However, single-cell analyses revealed major differences in the TME: adults had fewer CD4^+^ scTs, B-naïve and NK-cells, and more memory-B-cells. Cancer-associated-fibroblasts (CAF) with an Extracellular Matrix (ECM) deposition-signature (Matrix-CAFs) were more common in young mice, while pro-inflammatory stromal populations and myofibroblasts were more represented in adults. Matrix-CAFs in adult mice displayed decreased ECM-remodeling abilities, reduced collagen deposition, and a different pattern of interactions with the other cells of the TME. Taken together, our results suggest that age-dependent differences in the TME should be considered when designing preclinical studies.

## Introduction

The incidence of most cancer types increases significantly with age [1]. Prostate, colorectal, lung and pancreatic cancers are more common in older patients [2–5]. Likewise, the incidence of acute myeloid leukemia significantly increases in elderly people [6]. Although breast cancer can occur in younger women, it is more common in older ones [7]. These differences have been increasingly linked to aging of the TME [8], and to systemic age-related deficits of the immune system [9].

In the present era of cancer immunotherapy, there is increasing evidence that a tonic immune system is pivotal for the success of strategies to boost both the innate and the adaptive immune response against cancer cells [10]. This issue might be of most relevance for specific subsets of elderly patients, for instance those who have been previously exposed to therapies with side effects toxic to immune cells, such as some chemotherapeutics.

Despite this evidence, most preclinical studies are using very young mice of a few weeks of age as in vivo cancer models. While such mice have a fully functional, tonic immune system and thymus [11], the thymus undergoes several changes during aging that can profoundly affect T cells production and function, including thymic involution and related decline in diversity and quality of the T cell repertoire [12].

Our recent works have demonstrated that a Triple-Therapy (TT) involving antigen-presenting cell (APC) activation by vinca alkaloids and the generation of new TCF1^+^ stem cell-like T cells (scTs) by an alkylating agent can significantly enhance the efficacy of immune checkpoint blockers (ICB) in ICB-resistant cancer models such as triple negative breast cancer (TNBC) and Non-Hodgkin’s Lymphoma (NHL) [13–17]. We showed that TT efficacy was mediated by T cells, as it was abrogated by the in vivo depletion of CD3^+^CD4^+^ and/or CD3^+^CD8^+^ cells [14]. However, these models used young mice of a few weeks of age.

In this study, to address the discrepancies between the use of these cancer models in preclinical studies and cancer patient populations, we investigated differences in tumor growth kinetics, as well as TT efficacy and impact on immune and stromal cell populations in the tumor microenvironment (TME) of “young” (6–8 weeks of age) versus “adult” (12 months of age) mice, using preclinical cancer models of TNBC. Somewhat surprisingly, we identify a similar efficacy of TT in the two groups. Despite this, we pinpoint significant age-dependent differences in the TME, including in the ECM-remodeling ability of the cancer-associated fibroblasts (CAFs), which might be relevant when studying tumor growth and response to other therapies. Our results aim at bridging the gap between commonly used mouse cancer models and the challenges posed by more complex and heterogeneous populations of aging cancer patients.

## Results

### TT eradicates TNBC in young and adult mice

We have previously demonstrated in mouse models of TNBC and NHL that a novel Triple Therapy (TT, consisting of intermittent cyclophosphamide, anti-PD-1, and vinorelbine), is capable of halting both local and metastatic tumor growth [14, 17]. The efficacy of this therapy is dependent on T cells, as evidenced by its abrogation through in vivo depletion of CD3^+^CD4^+^ and/or CD3^+^CD8^+^ cells [14]. However, these earlier studies utilized 6 to 8-weeks-old mice, which inadequately mimic human cancer patient populations. Notably, 8 weeks in mice roughly correspond to human puberty [18]. However, TNBC onset in patients typically occurs around 30–40 years of age [19], which can be approximated to 12 months in mice [18]. To ensure consistency with human TNBC onset, we assessed the therapy’s efficacy in both “young” (6–8 weeks of age) and “adult” (12 months of age) mice. In both groups, we orthotopically injected 2 × 10^4^ 4T1-Luc cells, as previously performed [13, 14]. Young and adult mice underwent TT using the previously employed regimens [14, 17]. To confirm the expression of luciferase is maintained in vivo, we first measured the mRNA level of the transgene in tumors (see Methods). Non-luciferase-infected 4T1 cells were used as negative control, and in vitro cultured 4T1-Luc cells as positive control. qPCR showed that the expression is not only maintained, but increased in tumors as compared to cultured 4T1 cells (*p*-value < 0.0001, Student’s *t*-test; Supplemental Fig. S1A).

**Fig. 1:**
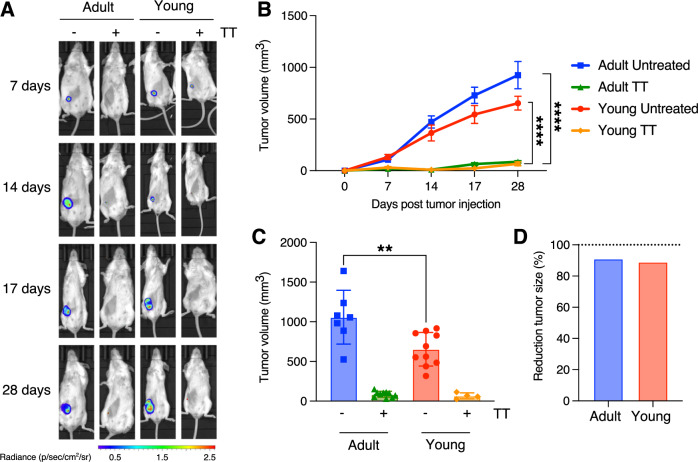
TT eradicates TNBC in young and adult mice. **A** In vivo live imaging of 4T1 tumors in adult (left side) and young (right side) mice treated either with vehicle or with TT. Images were taken at various time points, as indicated on the right. The luminescence/radiance bar on the left illustrates the intensity, with red corresponding to higher and blue the lowest luminescence. **B** Comparison of tumor growth in untreated and TT-treated young *vs.* adult mice (4T1 model; *n* = 10 per experimental group; ****: *p*-value < 0.0001, Student’s *t*-test). **C** Endpoint comparison reveals no differences in treated tumors, but a significant disparity in untreated tumors (with adult mice exhibiting larger tumors; **: *p*-value < 0.01, Student’s *t*-test). **D** Bar plot showing the average reduction in tumor mass upon TT for young and adult recipient mice.

As formerly demonstrated, TT effectively inhibits TNBC growth in young mice subjected to the therapy. Moreover, TT eliminates tumor growth in adult mice as well (Fig. 1A, B), supporting a similar efficacy of the therapy in adult subjects. However, we noted larger tumor masses developing in control adult mice compared to their young counterparts (Fig. 1C; *p*-value < 0.0001, Student’s *t*-test), resulting in a higher relative tumor regression (Fig. 1D). To eliminate any potential effects due to the cellular model, we extended our observations to another mouse TNBC model, EMT6, which was also employed in our previous studies [14, 16]. Also in this model, TT similarly and consistently inhibits tumor growth in both young and adult mice (Supplemental Fig. S1B).

### Single-cell transcriptomics highlight changes in the TME of adult mice

**Fig. 2:**
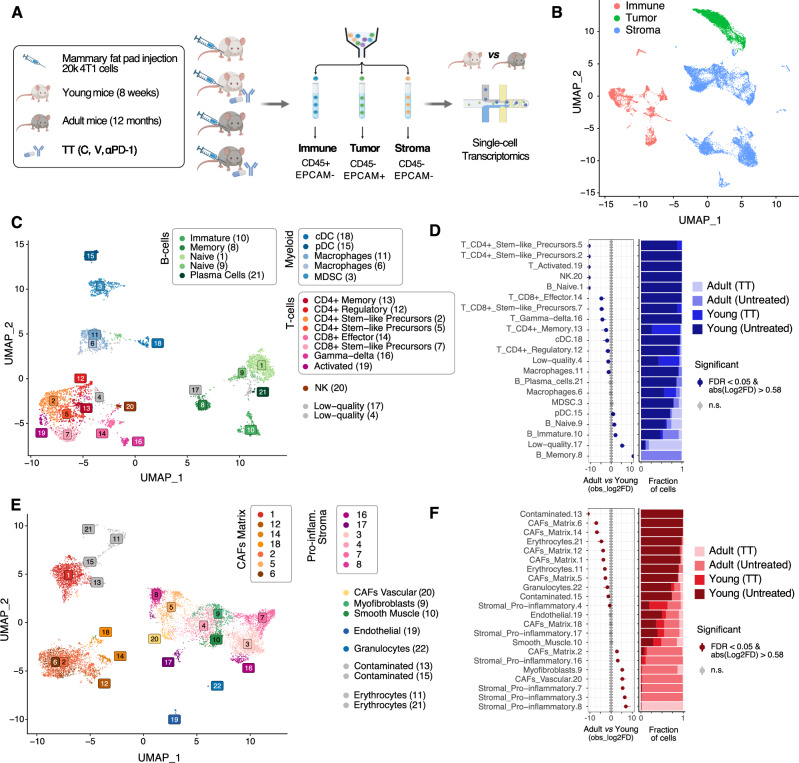
Single-cell transcriptomics highlight changes in the TME of adult mice. **A** Schematic of the experimental approach. Created with BioRender.com. **B** UMAP projection highlighting the overall structure of the single-cell RNA-seq data obtained. **C** UMAP projection of the filtered cells from the immune TME, color-coded by cluster. **D** (left) Relative differences in cell proportions for each immune TME cluster between cells from adult vs. young recipients. Clusters showing a significant difference (FDR < 0.05 and mean | Log2 fold enrichment | => 0.58) are highlighted (permutation test; *n* = 10,000). (right) Stacked bar chart showing the underlying cell composition of each cluster. **E** Same as **C** considering the cells from the stroma. **F** Same as **D** considering the cells shown in **E**.

These results prompted us to investigate whether a comparable efficacy of TT could be ascribed to a similar composition of the immune TME. To this end, we transcriptionally profiled 4T1 primary tumors (including TME cells) from both young and adult mice, either untreated or treated with TT, at the single-cell level, using single-cell RNA-sequencing (scRNA-seq) and consolidated the resulting data with previously published profiles (Fig. 2A, B; Supplemental Table S1). Briefly, we employed a FACS-sorting strategy to isolate tumor cells (CD45^-^EpCAM^+^), immune cells of the TME (CD45^+^EpCAM^-^), and stromal cells of the TME (CD45^-^EpCAM^-^) (Fig. 2A and Supplemental Fig. S1C, D) and subjected them to scRNA-seq using the 10x Genomics platform. We also included data from immune cells of the TME from young mice that we previously published [14, 16]. After consistent processing and filtering using a stringent computational pipeline (Supplemental Fig. S2A and Methods), we obtained a total of 23,694 high-quality cells across all captures (Supplemental Fig. S2B, C and Table S1). Initial clustering of cells from all three sorted populations ensured re-assignment of cells from each compartment (cancer, immune, and stromal cells) to the correct one, thus minimizing the effect of outliers from the FACS-sorting procedure (Fig. 2B and Supplemental Fig. S2B, C).

Cells were then clustered using the Leiden algorithm [20], separately for each compartment (tumor, immune TME, stromal TME). To define the optimal number of clusters in each of the three compartments, non-arbitrary thresholds were identified using the Silhouette score across an exhaustive range of resolutions (Supplemental Fig. S3A, B and Methods), as previously described [21]. This resulted in 21, 22, and 13 clusters of immune TME, stromal TME, and cancer cells, respectively (Fig. 2C, E and Supplemental Fig. S4A, and Supplemental Table S2). Cluster stability was confirmed by application of an orthogonal method (Supplemental Fig. S3C–E) [22].

The clusters in the immune and stromal TME were first automatically annotated using scType [23] and an extended database of marker genes for over 200 cell types (Supplemental Table S3). These annotations were manually refined, by comparing the computationally derived marker genes for each cluster (Supplemental Table S4) to manually curated sets of marker genes (Supplemental Table S3 and Supplemental Fig. S5A) showing high specificity for the cell types in the lineages identified by the automatic annotation. Considering the immune TME, all the major populations in the lymphoid and myeloid lineages were identified, including CD4^+^ and CD8^+^ scT cells (Fig. 2C and Supplemental Fig. S5A). In the stromal TME compartment, most cells displayed a signature of cancer-associated fibroblast (CAF) (Fig. 2E, Supplemental Fig. S5B–E, Supplemental Tables S2 and S4), along with distinct populations of endothelial cells, myofibroblasts, and smooth muscle cells (Supplemental Tables S2-4). Compared to previous studies in which several, distinct populations of CAFs were identified (Supplemental Table S4), in our dataset the majority of CAFs tend to show features of ECM-remodeling (termed matrix CAFs; Supplemental Fig. S5C-E) except for a small cluster of vascular CAFs (Supplemental Fig. S5D, E) expressing *Vegfa* and *Angpt1* (Supplemental Fig. S5G) [24]. We identified several CAF populations expressing an inflammatory signature (and lacking the ECM-remodeling signature) including e.g. the pro-inflammatory Macrophage Migration Inhibitory Factor *Mif* (together with other inflammatory markers, Supplemental Fig. S5F and Supplemental Table S4) [25] but lacking other markers previously associated with inflammatory CAFs [25, 26] (Supplemental Table S3 and Supplemental Fig. S5D). As compared to other studies performed so far [27], this might be related either to the different origin of the cancer-associated stromal populations, or to different interactions of these cells with the tumor cells, or to a different spatial location of these cells within the tumor niche.

As opposed to cells of the TME and, at least in part, as expected by 4T1 cells being less heterogeneous than primary tumors, cancer cells exhibited low heterogeneity. While it was possible to identify 13 clusters (Supplemental Fig. S3A, B), most cancer cells encompassed three clusters (clusters 1-3 accounting for 72.2% of the cancer cells; Supplemental Fig. S4A and Supplemental Table S2). All clusters showed only a limited number of marker genes (Supplemental Fig. S4B and Supplemental Table S4). At comparable levels of stringency, the three main clusters showed few to no distinctive marker genes, as compared to clusters identified in the immune or the stromal TME (Supplemental Fig. S4D, E). Also, none of the three major clusters exhibited a significantly altered representation in cells obtained from either young or adult donors (Supplemental Fig. S4C; FDR < 0.05 and |log2FD | > 1) [25].

We then tested whether cell type proportions in the TME were shifted based on age. Considering a stringent threshold (FDR < 0.05 and |log2FD | > 5) [25], the composition of the immune TME of young mice consisted of more T cells in general, while B cells were enriched in adult mice, with memory B cells exclusively occurring in adults and naïve B cells captured from both young and adult mice (Fig. 2D and Supplemental Table S5). While most myeloid cells did not show a population shift between young and adult mice, an inverse ratio of canonical vs. plasmacytoid dendritic cells (cDC vs. pDC) was observed at a lower stringency (FDR < 0.05 and |log2FD | > 0.58; Fig. 2D and Supplemental Table S5). Considering the stromal TME, using the same stringent thresholds applied to the immune TME, matrix CAFs showed a strong over-representation in the TME of young mice, as compared to that of adult mice. Pro-inflammatory stromal populations, myofibroblasts, and vascular CAFs were instead enriched in the TME of adult mice, as compared to the younger counterparts (Fig. 2F and Supplemental Table S5). These results prompted us to further leverage the obtained scRNA-seq profiles, with the aim of investigating the molecular, age-related differences in each of the cell types showing an altered representation.

### The immune TME composition of treatment-naïve tumors is influenced by age

**Fig. 3:**
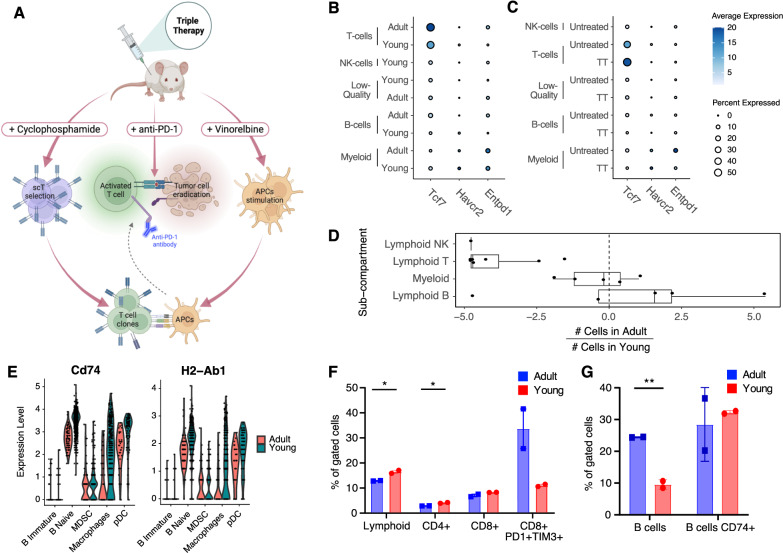
The immune TME composition of treatment-naïve tumors is influenced by age. **A** Graphical abstract depicting the preclinical effects of TT therapy on APCs, T cells, and cancer cells in TNBC models. Adapted from [13]. **B** Bubble chart highlighting no difference in the expression of the scT marker *Tcf1* (Tcf7) and exhaustion markers (*Havcr*2 and *Entpd1*) between adult and young immune TME cells (*q*-value > 0.05; Wilcoxon test). Size of the dot proportional to the fraction of cells in the indicated set with non-zero expression for the indicated gene. Color shade of the dot shows instead the average normalized expression of the gene in the indicated set. **C** Same as **B** but stratified by TT. **D** Box plots summarizing the bias in cell number (log2-fold-change) in adult *vs.* young immune TME, for the indicated sub-compartments (each dot is the result for a single cluster, Fig. 2D). Positive values indicate higher numbers of cells from adult mice in the cluster, negative values indicate higher numbers from young mice. **E** Representative genes involved in antigen presentation showing downregulation of expression in the adult *vs.* young B cells and macrophages of the TME (*q*-value <= 0.05; Wilcoxon test). **F**, **G** FACS validation of the biases shown in panel D (*: 0.01 <= *p*-value < 0.05, **p-value < 0.01; Student’s *t*-test), Five mice were pooled in each experimental group (*n* = 2).

In order to fully extend the previously described mechanism of action of TT [14] (Fig. 3A) to tumors in adult mice, we first aimed at verifying that the major, relative differences in T cell abundance (and scT abundance) between the TME of adult vs. young mice (Fig. 2D) were not reflected also in major age-related differences in the transcriptional profile of cells of this lineage. T cells showed a similar profile in adult and young lesions, with *Tcf7* (TCF1), and *Havcr2* and *Entpd1* (markers of exhaustion) showing no significant difference in expression (Fig. 3B and Supplemental Fig. S6A; *q*-value > 0.05; Wilcoxon test). As previously shown in young mice [14], this supports the idea that the efficacy of TT is mediated by stem-like progenitor T cells also in adult mice. Of note, these markers also showed similar expression patterns after TT (Fig. 3C), despite the low residual number and inferior quality of cells captured from the immune TME of TT treated tumors (Supplemental Fig. S2C).

We then further aggregated the results from the differential abundance analysis (Fig. 2D) by major immune lineage (B, T, NK, myeloid). This showed that the myeloid compartment was the least affected by age, and further highlighted that B and T are the most affected compartments, with some B cell clusters being an exception (Fig. 3D). To gain further insight into the molecular, age-related differences between cells from young vs. adult TME, per cell type, we ran a differential gene expression analysis, followed by an enrichment analysis using KEGG [28] and the Hallmark gene sets [29] (see Methods). This highlighted a major over-representation of genes related to antigen presentation pathway (adjusted *p*-value <= 1e-9; hypergeometric test) in genes down-regulated in B cells and macrophages from the TME of adult mice (Supplemental Fig. S6C and Supplemental Table S6). This signal was driven by many differentially expressed genes in the pathway (Supplemental Table S6), including *H2-Ab1* and *Cd74* (Fig. 3E). This is in line with a recent study which also showed down-regulation of antigen-presentation in aged TME of melanoma and lung cancer models [30].

To validate these results at the protein level, we subjected 4T1 tumors of independent young and adult mice to flow cytometry, using a panel of markers to quantify both T and B cells, and CD74 (Fig. 3F). Consistent with the transcriptomic analysis, the TME of young mice exhibited an enrichment in the lymphocyte population (*p*-value < 0.05, Student’s *t*-test), particularly in the CD3^+^CD4^+^ subpopulation (*p*-value < 0.05, Student’s *t*-test). The same trend, though not statistically significant, was observed in the CD3^+^CD8^+^ subpopulation, with the CD8^+^PD1^+^TIM3^+^ population being more abundant in adult mice (Fig. 3F; *n* = 2, *p*-value > 0.05, Student’s *t*-test). In parallel, we assessed B cell abundance through CD19^+^ staining. Also in this case, the results at the protein level confirmed the observation at the transcriptional level, revealing an overall increased number of B cells in the TME of adult mice, compared to their younger counterparts (*p*-value < 0.01, Student’s *t*-test). Conversely, we observed a trend for CD74 expression to be heightened in the B cells of young mice (Fig. 3G). Immunohistochemistry (IHC) stainings were then conducted to quantify CD3^+^CD4^+^, CD3^+^CD8^+^, CD19^+^, and FOXP3^+^ cells. This confirmed the age-related trends observed in 4T1 tumors via scRNA-seq and flow cytometry (Supplemental Fig. S7A), and indicated similar in EMT6 tumors, except for the CD3^+^CD4^+^ subpopulation (Supplemental Fig. S7B). This discrepancy may be attributed to intrinsic differences in the immune TME of these models of TNBC, as we previously reported [14, 16]. To further investigate the localization of immune cells within the tumor, we performed double immunofluorescence (IF) staining, labeling immune cells and tumor cells at the same time. These showed immune cells are interspersed within the tumor, with lack of specific localization, in both 4T1 and EMT6 (Supplemental Fig. S7C–F).

These results prompted us to also investigate whether at least part of the age-related differences in the abundance and transcriptional profile of the TME subpopulation were already detectable in the immune cells of the ageing mammary gland. To this end, we re-analyzed and mined single-cell transcriptomics data from *Tabula Muris Senis* (TMS) [31], using an approach in line with that applied to the tumor data (see Methods). Cells from 3-months old mice were deemed as “young”, while those from 18- or 21-months old mice as “old”. Using two distinct optimal resolutions for clustering (Supplemental Fig. S8A–F and Supplemental Table S7) robustly showed that in the aged mammary gland some populations of T cells tend to be significantly under-represented (FDR < 0.05 and |log2FD | > 0.58), while the population with the strongest over-representation in old mice is one of B cells (Supplemental Fig. S8G–I and Supplemental Table S7). Nevertheless, no major differences in the expression of antigen presentation genes were identified in such population (Supplemental Table S7I). These observations suggest that at least part of the observed age-related differences in the immune cell of TME might reflect an involution process that is already happening during the ageing of the mammary gland.

Taken together, these results support the existence of age-related differences in the immune TME of treatment-naïve TNBC. While these do not seem to affect the response to TT, they might affect the efficacy of other therapies in these preclinical models, depending on which cell populations drive the anti-tumor response.

### Stromal TME cells show age-related, pro-tumorigenic features

**Fig. 4:**
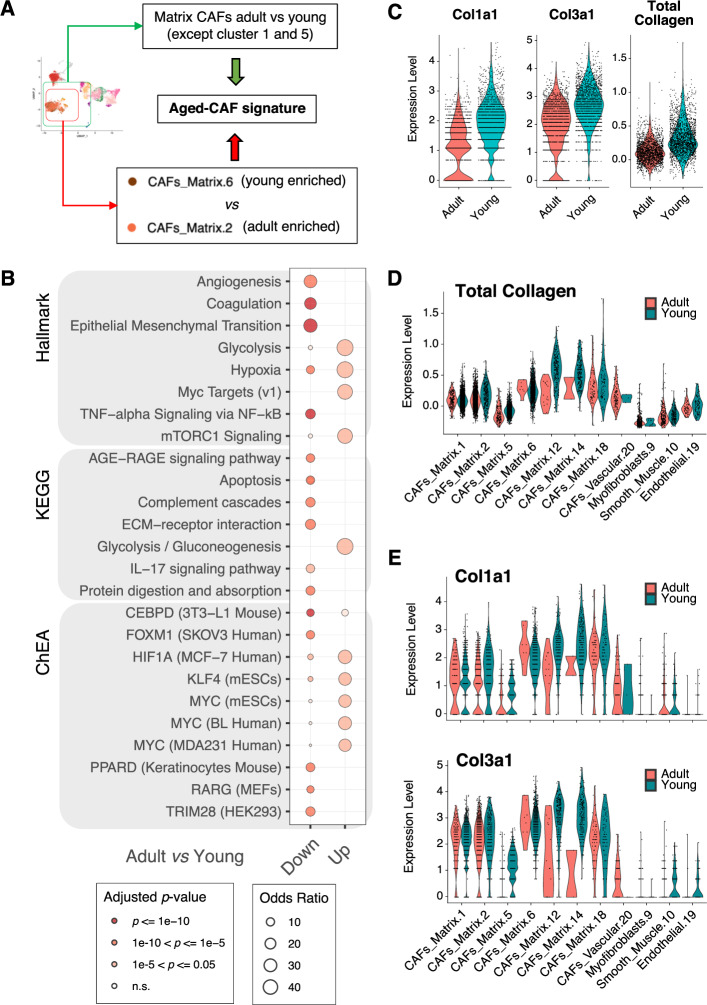
Stromal TME cells show age-related, pro-tumorigenic features. **A** Schematic showing how the transcriptional signature of aged CAFs was derived. **B** Bubble plot summarizing the results of functional enrichment analysis (using the hallmark gene sets, the KEGG pathways, and the ChEA regulons) of the up- and down-regulated genes in the aged-CAF signature in **A**. Size of the bubble proportional to the odds ratio; color-coded based on statistical significance of the enrichment (adjusted *p*-value; hypergeometric test). **C** Violin plots showing the expression of selected collagen genes (*Col1a1* and *Col3a1*) or all collagen genes (total collagen) in single CAFs from the TME of either adult or young mice. **D** Violin plots showing the total collagen expression in single cells of selected clusters, split by age group of the recipient. **E** Same as **D** for *Col1a1* and *Col3a1*.

Based on our initial observation that matrix CAFs are enriched in the TME of young mice (Fig. 2F), and the most abundant populations in the stromal TME (Fig. 2E), we further investigated the molecular features potentially driving the age-related differences in these CAFs. To this aim, we first focused on the largest matrix CAFs clusters [1, 2, 5, 6]. Whereas cells from cluster 2 were predominantly derived from adult mice, cluster 6 was enriched for TME of young mice (Fig. 2E, F). These two clusters displayed significant differences in collagens type I and III expression (Supplemental Fig. S9A). We therefore ran a differential expression analysis between the cells in these two clusters. This quantitatively confirmed the gene expression trend of collagen genes (Supplemental Table S8; including but not limited to *Col1a1-2*, *Col3a1*, *Col5a1-2*; *q*-value < 0.05; Wilcoxon test). We also confirmed that similar results could be obtained comparing the total matrix CAFs from TMEs of adult mice vs. young mice, irrespective of their cluster (Supplemental Table S8, Supplemental Fig. S5B and Methods). To then derive a robust signature of age-related differences of matrix CAFs, we retained all genes common to the two signatures described above and showing a consistent direction of change (Fig. 4A, Supplemental Table S8 and Methods). We then identified enriched pathways in the young (down-regulated) and adult (up-regulated) aged-CAF signature using KEGG [28], the Hallmark gene sets [29], and the ChEA regulons [32]. The results highlighted the down regulation of processes related to the remodeling of the ECM, and the up regulation of metabolic and hypoxic pathways, in CAFs from the TME of adult mice (adjusted *p*-value <= 1e-5; hypergeometric test; Fig. 4B and Supplemental Table S8). Among the down-regulated genes related to a different ECM-remodeling ability, we identified an enrichment of genes encoding for glycoproteins, basement membrane components, and collagens (Supplemental Table S8). Age-dependent transcriptional down-regulation of collagens type I and III, as well as of total collagen, were assessed and confirmed at both global (Fig. 4C) and individual cluster level (Fig. 4D, E). Collagen I downregulation was previously reported in aged skin fibroblasts [33]. This correlation prompted us to leverage the re-analyzed single-cell transcriptomics data from TMS [31] (Supplemental Fig. S8 and Supplemental Table S7). Indeed, both *Col1a1* and *Col3a1* showed significant down-regulation in stromal cells of normal mammary glands of old mice compared to young ones, in four (out of six) individual clusters (adjusted *p*-value <= 0.05; Wilcoxon test; Supplemental Fig. S8M) and overall (Supplemental Table S7I). The age-related CAF signature we derived (Fig. 4B and Supplemental Table S8) instead showed only a minimal overlap with the age-related differences in clusters of stromal cells in the ageing mammary gland (Supplemental Fig. S8N–O and Supplemental Table S7), with down-regulated genes in old cells showing statistically significant, but quantitatively small enrichments (adjusted *p*-value <= 0.05; hypergeometric test; Supplemental Table S7L). These results suggest that transcriptional differences in fibroblasts normally occurring during ageing of the mammary gland might be exacerbated in the presence of tumor cells.

Remarkably, most of the gene sets significantly associated with the age-related CAF signature (Fig. 4B and Supplemental Table S8) correlate with higher pro-tumorigenic ability of the CAFs in adult vs. young mice [24]. This is in line with the larger tumor size observed in adults, as compared to young mice (Fig. 1B, C).

Interestingly, when assessing statistically significant differences in the abundance of stromal cells derived from TME of TT vs. treatment-naïve tumors, we identified a cluster with a pro-inflammatory signature (cluster 4) showing a significant bias towards TT. Surprisingly, this is not among those pro-inflammatory populations with a strong bias for the adult TME, but it is well represented in TME of both young and adult mice (Supplemental Fig. S9B; FDR < 0.05 and |log2FD | > 1) [25].

### CAFs in the TME of tumor of adult mice display altered ECM-remodeling features

The results obtained from single-cell transcriptomics experiments prompted us to further validate the observed age-related differences in CAFs abundance, and in their ECM-remodeling abilities, in orthogonal samples, in their native context.

**Fig. 5:**
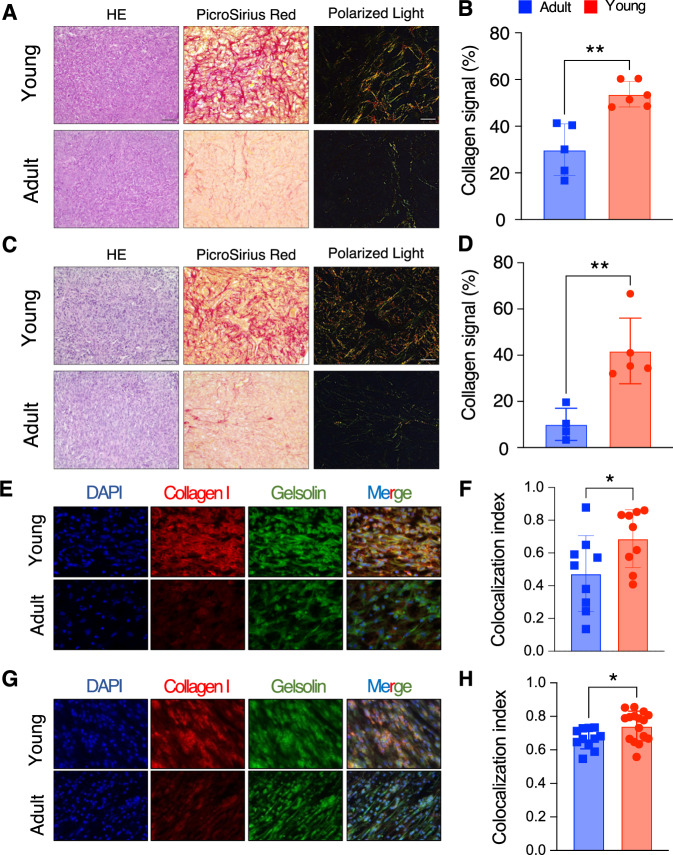
CAFs in the TME of tumor of adult mice display altered ECM-remodeling features. **A** HE, Picro-Sirius Red and Polarized light in one representative young (top panel) and old (bottom panel) mice for the 4T1 model. Scale bar shown in the HE panel, with the bar equal to 50 μm. **B** Quantification of Picro-Sirius Red staining over the tumor surface (*n* = 6 adult and *n* = 5 young mice) for the 4T1 model shown in **A**. **C** Same as **A**, for the EMT6 model. Scale bar shown in the HE panel, with the bar equal to 50 μm. **D** Quantification of Picro-Sirius Red staining over the tumor surface (*n* = 5 adult and *n* = 4 young mice) for the EMT6 model shown in **C**. **E** Collagen I and gelsolin IF in one representative young (top panel) and adult (bottom panel) mice for the 4T1 model. The first panel from the left shows DAPI in blue, the second collagen I in red, the third one gelsolin in green, and the fourth one the merged signals between the three different stainings. **F** Colocalization index, calculated as overlap between collagen signal (in red) and gelsolin signal (in green) from IF data from 4T1 (five independent fields derived from three mice per experimental conditions). **G** Same as **E**, for the EMT6 model. **H** Same as **F**, for the EMT6 model. (**B**, **D**, **F**, **G**; *: 0.01 <= *p*-value < 0.05, ** *p*-value < 0.01; Student’s *t*-test).

First, we measured collagen abundance in tumor slides, using Picro-Sirius Red staining, which allows to evaluate the overall collagen levels (types I and III) within tumors. Collagen abundance was assessed in five different mice per condition (young/adult) (Fig. 5A for representative slides). In line with scRNA-seq estimates (Fig. 4C–E) in stromal cells, collagen was found to be significantly more abundant in young compared to adult mice [14, 16] (Fig. 5B; *p*-value < 0.01, Student’s *t*-test). To rule out any effect arising from the specific cellular model (4T1), we extended our observations to another TNBC model (EMT6), which we showed to also respond to TT in an age-independent fashion (Supplemental Fig. S1A).

Of note, the overall collagen abundance in this model was also higher in young mice compared to older ones (Fig. 5C, D; *p*-value < 0.01, Student’s *t*-test), strongly suggesting that the difference arises from the TME rather than the specific cellular models employed. Moreover, staining with Picro-Sirius Red can be leveraged to highlight the inherent birefringence in collagen fibers when exposed to polarized light. This method can be used to assess the structural organization of collagen fibers within tissues. When examining tumor slides from young mice stained with Picro-Sirius Red under polarized light, thick collagen type I fibers exhibit shades of yellowish-orange, orange, or red. In contrast, when observing tumor slides from adult mice, thinner collagen type III fibers appear green to yellowish-green against a black background (Fig. 5A, C).

To confirm that this altered ECM deposition in adult vs. young mice is mediated by CAF subtypes identified in our transcriptomic data we performed immunofluorescence (IF) on tumor slides (obtained from a subset of mice from the previous sets of experiments, i.e., either used in scRNA-seq, FACS, or IHC) targeting both collagen I and gelsolin (*Gsn*), a gene we found to be strongly expressed in matrix CAFs, in our scRNA-seq data (Supplemental Fig. S9C). First, IF analysis on collagen I confirmed a consistently lower expression in the TME of adults as compared to that of young mice, in both cellular models (4T1 and EMT6; Fig. 5E, G). Furthermore, to precisely estimate collagen I exclusively released by CAFs, we conducted double IF staining for collagen I and gelsolin. Remarkably, gelsolin expression exhibited a strong correlation with collagen I expression (Fig. 5E), with a higher colocalization index in young mice compared to older ones (Fig. 5F), indicating increased collagen I expression by CAFs in young mice. This analysis was also extended to EMT6 tumor slides, yielding comparable results (Fig. 5G, H).

Taken together, these results further corroborate major differences in the infiltrated matrix CAFs in the TME of adult vs. young mice, both numerically and in a different capacity of CAFs to (1) deposit collagen and (2) contribute to structural differences in the ECM. These results confirm our initial observation based on measurements at the gene expression level alone. Taken together, these results shed light on notable and clinically relevant differences in stromal cell populations in young vs. adult murine models. These might be important in preclinical studies of therapeutic efficacy, depending upon the cell populations driving the specific anti-tumor response.

### Interactions between CAF, myeloid and endothelial cells in the TME are subject to aging

**Fig. 6:**
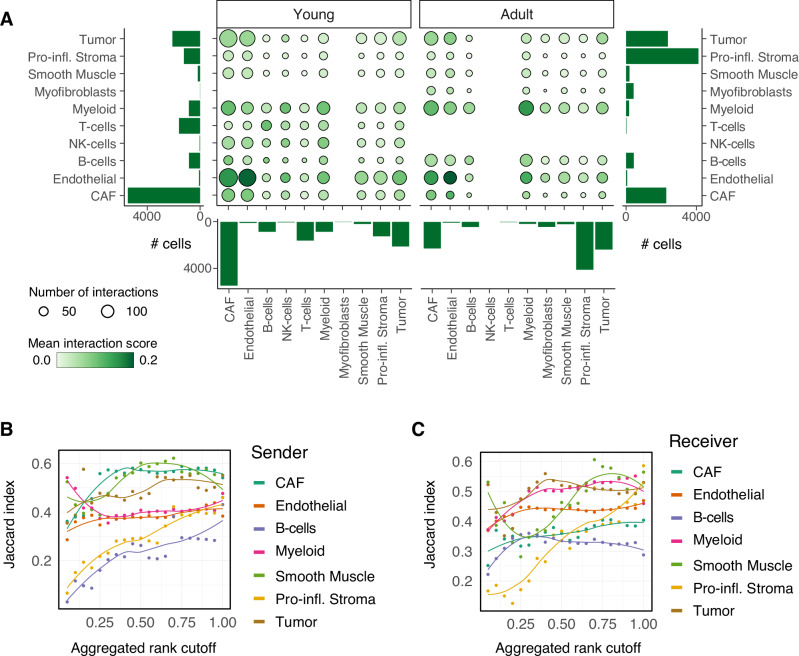
Cell-cell communication results. **A** Bubble plot summarizing the mean interaction scores (specificity * sensitivity, color of the bubbles) and numbers of predicted interactions (size of the bubbles) across pairs of cells populations grouped by the indicated sub-compartments, separately in young and adult mice. Bar charts indicate cell numbers per group. **B**, **C** Similarity (average Jaccard index) between the predicted interactions in young and adult mice at increasingly stringent thresholds on the aggregated rank across all pairs, when considering the indicated sub-compartments either as **B** sender and **C** receiver. Individual data points along with a smoothing curve fitted by loess regression are shown.

The observed age-dependent differences in the abundance and characteristics of CAF populations in the TME raised the question of whether and how these changes might influence intercellular interactions among different cell populations. We therefore applied ligand-receptor analysis, using a combination of computational methods and their consensus results, implemented in the LIANA framework [34], separately on young and adult cell populations. First, we compared general patterns (the number and the relevance) of cell-cell communication predictions and found that overall, both age groups share a high similarity in their interactions (Fig. 6A, Supplemental Fig. S10A, B, D and Supplemental Table S9). We confirmed this observation quantitatively by assessing similarity between the interactions of ligand-receptor pairs for young and adult mice at increasingly lenient thresholds of significance of the interaction predictions (Fig. 6B, C, Supplemental Fig. S10C, E, F; based on Jaccard index, see Methods). All populations showed very similar patterns of interactions with the other cell types between young and adult mice, except for predictions in the smallest cell populations, at lower stringency (Fig. 6B, C).

Digging into the strongest interactions between individual cell populations corroborated these observations, according to which most interactions occur both in young and adult mice (Supplemental Fig. S11A–F and Supplemental Table S9), with a few exceptions. Predictions indicate that CAF populations preferentially signal to myeloid cells in the TME of adult mice, via specific ligand-receptor pairs that include the complement system and integrins. In turn, myeloid cells show altered signaling to endothelial cells between young and adult mice, for example through the ligand Pecam1, which appears to be exclusively in the prediction of the adult mice (Supplemental Fig. S11D and Supplemental Table S9).

Taken together, these results suggest that while differences in the TME of young and adult mice are larger in terms of abundance and gene expression profile of the different cell populations, an age-specific axis of interaction between CAF, myeloid, and endothelial cells might exist.

## Discussion

Hallmarks of aging that are relevant for cancer progression and response to therapy include: (a) an increase in systemic low-grade chronic inflammation; (b) a widespread change in gene expression, resulting in increased secretion of proinflammatory cytokines, chemokines, growth factors, and proteases; (c) an age-related decrease in the number and proliferation of healthy stromal cells; (d) an increase of pro-tumorigenic Myeloid-Derived Suppressor Cells (MDSC); (e) an increase in pro-tumorigenic Tregs; and (f) a remodeling of the collagen matrix promoting cancer metastasis and affecting immune cell motility [8, 11, 35]. Collectively, these imbalances have been recently described as “Age Related Immune Dysfunction” (ARID) [36].

Despite this, currently used cancer rodent models usually employ very young animals, and frequently their preclinical efficacy data do not predict clinical outcome in cancer patients [37]. These discrepancies generate frustrating losses of time, money and hope in cancer patients. In this context and considering that most TNBC patients are adults, the aim of the present study was to elucidate whether: (i) TNBC local and metastatic growth; (ii) TT preclinical activity; and (iii) the TME, including intra-tumoral immune and stroma cells, did differ in young (6–8w) versus adult (12 m) immune competent mice.

**Fig. 7:**
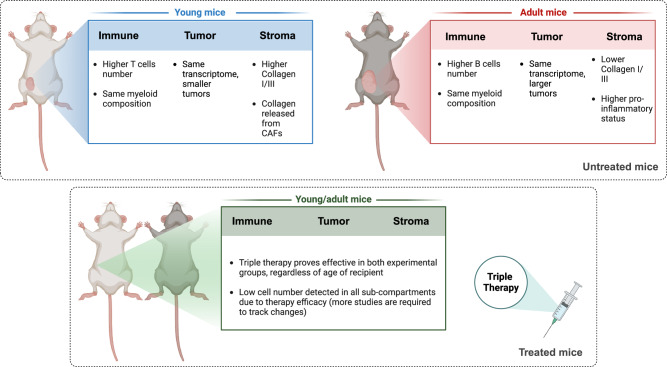
Schematics of the findings. Schematic representation describing cell populations and enriched pathways in untreated young *vs.* adult mice (top panel) as well as TT-treated young *vs.* adult mice (bottom panel). Created with BioRender.com.

As summarized in Fig. 7, single-cell profiling showed that despite similar efficacy of TT in young and adult mice (Fig. 1), the TME of these tumors differed considerably in relation to age (Fig. 2). We identified differences in the composition of both immune cells infiltrating the tumor (Fig. 3) and in the cancer associated fibroblasts (Figs. 4–6). Matrix CAFs were more common in young mice, and pro-inflammatory stromal populations in adults. Matrix-CAFs displayed decreased ECM-remodeling abilities, reduced collagen deposition, and a different pattern of interactions with the other cells in the TME in adult mice (Fig. 6 and Supplemental Fig. 9).

On one hand, TT efficacy was similar in young and adult mice, as the treatment abrogated local and metastatic growth (Fig. 1A–C). This result is in line with our present observation that CD8^+^ (but not CD4^+^) scTs (Fig. 2D), were only slightly reduced in adult mice despite reported age-related thymus involution [12]. Available data also suggests that CD8+ scTs cells might egress to the draining lymph nodes, which could potentiate systemic response and contribute to long-term maintenance of the compartment, irrespective of the different abundance of the infiltrated CD8+ scTs in the TME of young vs. adult mice [38].

On the other hand, single-cell RNA-seq, IHC and flow cytometry analyses of TME immune (CD45^+^EpCAM^-^) and stromal (CD45^-^EpCAM^-^) cell populations indicated major differences in young vs. adult mice (Figs. 2C–F, 3, 4 and 5). Adults had significantly less CD4^+^ scTs, B naïve and NK cells and significantly increased memory B cells (Figs. 2D, 3D, F, G and Supplemental Fig. S7A, B). These changes are in line with what has been observed in recent studies investigating the aged TME in different models [30, 39]. In addition to significant differences in the immune TME, we describe profound differences in the stromal TME compartment. One of the major observations of our study is that matrix CAFs were more represented in the TME of young mice, while pro-inflammatory stromal populations and myofibroblasts were more abundant in that of adult mice (Figs. 2F, 4 and 5). Moreover, matrix CAFs from adult mice displayed down-regulation of a signature involved in different ECM-remodeling abilities, and up-regulation of metabolic and hypoxic pathways (Fig. 4B). Expression of genes encoding for glycoproteins, basement membrane components, and collagens were also upregulated (Fig. 4C–E and Supplemental Table S8). Notably, these observations could be extended at the protein and cellular level (Fig. 5), with matrix CAFs from TME of young mice being more proficient in depositing collagen (Fig. 5E–H) and giving rise to a more complex network of thicker collagen fibers (Fig. 5A–D). Interestingly, the molecular differences identified at the transcriptional level in CAFs from TME of adult mice, as compared to that of young mice (Fig. 4B and Supplemental Table S8) correlate with higher pro-tumorigenic features of the CAFs in adult vs. young [24]. This evidence was further supported by the cell-cell interaction predictions (Fig. 6 and Supplemental Fig. S9). These suggest that most of the interactions between myeloid and pro-inflammatory stromal cells in adults involve recruitment and activation of myeloid cells. We speculate that this contributes to a pro-inflammatory TME in the adults, which could explain the observed increased tumor size (Fig. 1B, C). Our results in adult vs. young CAFs are in line with previous observations in fibroblasts from the aging skin, showing reduced collagen type I and III expression, and increased pro-adipogenic features [33], as well as with a recent study showing that CAFs in the aged TME can promote pancreatic cancer growth and progression [40]. Furthermore, a recent report highlighted a similar correlation between pro-inflammatory, adipose-enriched features of the peri-tumoral TME and lower survival, in breast cancer [32]. In line with this, collagen was previously found to be negatively correlated with a pro-inflammatory status [41, 42]. Thus, we can speculate that a decrease in collagen deposition may be also associated with a higher inflammatory state, which could explain the increased tumor growth observed in the adults. At the same time, while age-related differences in the ECM affecting cancer initiation, progression, and metastasis have been documented, they seem to be tumor tissue-specific and depending on the initial tumor stiffness [35, 43]. This further highlights the value of considering the age of the animals as a critical factor when designing pre-clinical studies.

Despite limited data at the single-cell level being available [44, 45], we sought to mine published single-cell transcriptomics profiles from primary, human TNBC that included subjects in different age groups, using the signature of aged matrix CAFs we defined (Fig. 4A). The results showed inconsistent results (Supplemental Fig. S12A, B), suggesting that larger cohorts are required to consistently extend our results to human data. In one dataset [44] we could validate the up-regulated genes of our signature, but not the down-regulated ones. In the other one [45] we could find a consistent, statistically significant difference in the down-regulated axis of the signature, but not the up-regulated one. While using the entire signature returned inconsistent results, random forest classifiers trained independently on the two datasets were able to distinguish CAFs based on the age of the donor, with high sensitivity and specificity (>90%; Supplemental Table S12A), based on a consistent subset of genes (Supplemental Fig. S12C and Supplemental Table S12B, C).

One major limitation of the present study is a lack of measurements considering the structure of the cancer tissue, especially providing spatial information about the TME. This limitation will be addressed in the future by coupling spatial transcriptomics and high-throughput IHC with scRNA-seq technologies. Another limitation of this study is the resolution at which different subpopulations of T cells, B cells, and myeloid cells of the TME of both treated and treatment-naive mice have been profiled. Recent studies suggest age-dependent differences in the abundance of subpopulations of CD8+ scT cells [30] and atypical B cells [46]. Enrichment strategies along with profiling of cells from the draining lymph nodes, will be required to deepen the resolution of our findings, and identify more fine-grained, age-related differences. This notwithstanding, our data indicate profound differences between young and adult mice immune and stromal TMEs that should be taken into consideration when selecting the age of mice to be used in preclinical efficacy studies in orthotopic immune competent mice. The efficacy of TT depends on CD8+ scT cells, and our data indicate that these cells are transcriptionally indistinguishable in adult and young TMEs (Fig. 3B, C and Supplemental Fig. S6A–C), with their frequency only minimally affected by age (Figs. 2D, 3F–G, and Supplemental Fig. S6D, E). Conversely, if a given anti-cancer immunotherapy relies on CD4+ scT cells, B naïve, or NK cells, age of murine models should be more carefully considered to represent the immune TME of the human patients. Along a similar line, when assessing the preclinical efficacy of therapies targeting the TME (e.g., anti-angiogenic, or anti-CAF therapies), and particularly in tumors surrounded by dense stroma such as pancreatic cancer [45], selection of mice with an age representative of the age of human patients to be treated will be paramount. Large scale studies with spatial resolution in age-adjusted models, that consider young, adult, and even older cohorts, are required to address these questions.

## Materials and methods

### Cell culture

The 4T1 and EMT6 cell lines were purchased from ATCC, expanded, and stored according to the producer’s instructions. Cells were tested by means of PCR to detect any different Mycoplasma strains, cultured for no more than 2 weeks, and used for no longer than 15 passages. Cells were stably infected with a vector expressing luciferase as previously shown [14].

### Gender bias

Our study exclusively examined female mice because the disease modeled is only relevant in females.

### In vivo studies

Experiments involving animals were approved by the Italian Ministry of Health and have been done in accordance with the applicable Italian laws (D.L.vo 26/14 and following amendments), the Institutional Animal Care and Use Committee, and the institutional guidelines at the European Institute of Oncology, following ARRIVE guidelines (projects 267/2020 and 219/2024). In vivo studies were carried out in 6 to 8 weeks and 12 months old immune-competent BALB/cOlaHsd female mice (Envigo) in mouse facilities at the European Institute of Oncology - Italian Foundation for Cancer Research (FIRC) Institute of Molecular Oncology (IEO-IFOM, Milan, Italy) campus. To generate syngeneic models of TNBC in BALB/c, 2 × 10^4^ 4T1-LUC and 5 × 10^4^ EMT6-LUC were injected in the mammary fat pad as we described previously [13, 14]. Tumor growth was monitored weekly using In Vivo Imaging System (IVIS; PerkinElmer). Briefly, mice were intraperitoneally injected with 150 mg/kg of XenoLight D-Luciferin-K+ Salt Bioluminescent Substrate (PerkinElmer, # 122799). After 10 min, animals were anesthetized with isoflurane apparatus and images acquired using Living Image Software (PerkinElmer). Tumor dimension was finally assessed using ImageJ software, by converting pixel intensity into centimeters and finally determining the tumor mass sphere volume in mm^3^. The experiments were performed in a blinded manner by two operators: the first was responsible for injecting luciferin intraperitoneally (IP), while the second took the measurements using IVIS, without knowledge of the experimental groups.

### In vivo therapy

Young and adult tumor-bearing mice (*n*  = 10, per study arm) were treated with either vehicle or triple therapy 3 days after tumor implantation to ensure similar tumor sizes, as done previously [14]. Triple therapy comprises three drugs: Cyclophosphamide, Vinorelbine and anti-PD-1. Cyclophosphamide (C) at 140 mg/kg, Vinorelbine (V) was used at 9 mg/kg as we have previously shown [13, 14, 17]. Chemotherapeutic drugs were dissolved in PBS and administered, once a week, every 6 days, for 3 weeks by intraperitoneal injection. Mouse monoclonal PD-1-targeting (Bioxcell, #BE0146, clone RMP1-14, AB_10949053) and was administered intraperitoneally 0.2 mg/mouse every 2 days for a total of five doses.

### Luciferase mRNA expression quantification

To monitor luciferase expression in vivo, quantitative Real Time PCR was performed on tumor-sorted cells (EpCAM + ). Briefly, three mice were injected with 20,000 4T1-Luc. Tumors were surgically removed after 28 days from tumor injection to generate a single cell suspension. Briefly, after mechanical dissociation, tumors were placed in culture medium (1:1 of DMEM with high glucose and Ham’s F-12 Nutrient Mixture; EuroClone) supplemented by 2 mg/mL collagenase (Merck, #SCR103) and 0.1 mg/mL Dnase I (Qiagen, #79254), and digested for 1 to 2 h at 37 °C. A single cell suspension was obtained by sequential dissociation of the fragments by gentle pipetting, to further disintegrate cell clumps, followed by filtration through a 100-μmol/L nylon mesh. After cell suspension preparation, cells were incubated with murine PeCy7 EpCAM (Biolegend, #118216, clone G8.8, RRID AB_1236471) for 20 min at 4 °C. Cells were then washed twice with PBS + 0.2% BSA and prepared for cell sorting. Before sorting, cells were co-stained with 0.1 μg/mL 4′,6-diamidino-2-phenylindole (DAPI, Merck #D9542). Cells were sorted by FACS Fusion sorter (BD Bioscience) for tumor cells EpCAM^+^CD45^-^DAPI^-^ and pelleted. RNA was then extracted from cell pellets using RNeasy® Micro kit (Qiagen) following manufacturer’s instructions. 1 µg of RNA was retro transcribed using High-Capacity cDNA Reverse Transcription Kit (Thermofisher). For gene expression analysis, 5 ng of cDNA was amplified (in triplicate) in a reaction volume of 10 µL containing the following reagents: 5 µl of “TaqMan® Fast Advanced Master Mix, Thermofisher”, 0.5 µl of TaqMan Gene expression assay 20x, (Thermofisher) (for details see the list below). Real-time PCR was carried out on the 7500 Real-Time PCR System (Thermofisher), with the following settings: (1) 20 s at 95 °C; (2) 40 cycles of 1 s at 95 °C, followed by 20 s at 60 °C. Raw data (Ct) were analyzed with Biogazelle Gbase Plus software and the fold change was expressed as CNRQ (Calibrated Normalized Relative Quantity) with Standard Error (SE). GeNorm Software chose Gapdh and Hprt1 as best housekeeping genes and the geometric mean of these two genes was used to normalize the data. Taqman assays were performed using the following reagents: Luc assay ID: Mr03987587_mr; Gapdh assay ID: Mm99999915_g1; Hprt1 assay ID: Mm00446968_m1.

### Tumor dissociation and cell sorting for scRNA-seq library preparation

Tumors were surgically removed after 28 days from tumor injection to generate a single cell suspension. Briefly, after mechanical dissociation, tumors were placed in culture medium (1:1 of DMEM with high glucose and Ham’s F-12 Nutrient Mixture; EuroClone) supplemented by 2 mg/mL collagenase (Merck, #SCR103) and 0.1 mg/mL Dnase I (Qiagen, #79254), and digested for 1 to 2 h at 37 °C. A single cell suspension was obtained by sequential dissociation of the fragments by gentle pipetting, to further disintegrate cell clumps, followed by filtration through a 100-μmol/L nylon mesh. After cell suspension preparation, cells were incubated with PE-conjugated murine CD45 antibody (BD Biosciences, #553081, clone 30-F11, RRID AB_394611), murine PeCy7 EpCAM (Biolegend, #118216, clone G8.8, RRID AB_1236471) for 20 min at 4 °C. Cells were then washed twice with PBS + 0.2% BSA and prepared for cell sorting. Before sorting, cells were co-stained with 0.1 μg/mL 4′,6-diamidino-2-phenylindole (DAPI, Merck #D9542). Cells were sorted using different gating strategies using FACS Fusion sorter (BD Bioscience): EpCAM^-^CD45^+^DAPI^−^ for immune cells, EpCAM^+^CD45^-^DAPI^-^ for tumor cells and EpCAM^-^CD45^-^DAPI^−^ for stromal cells (Supplemental Fig. S1B and C). Antibodies are listed in Supplemental Table S10. Cells were then counted, and 10,000 cells/conditions underwent scRNA-seq library preparation following 10X Genomic V3.1 protocol.

### Immunohistochemistry and Immunofluorescence staining

Mouse tumors were fixed in 4% paraformaldehyde PFA and processed by a Diapath automatic processor as follows. Tissues were dehydrated through 70% (60 min), 2 changes of 95% (90 min each), and 3 changes of 99% (60 min each) ethanol, cleared through 3 changes of xylene (90 min each), and finally immersed in 3 changes of paraffin, 1 h each. Samples were embedded in a paraffin block and stored at room temperature until ready to section. According to standard protocol, Haematoxylin/Eosin (Diapath) and Picro-Sirius Red staining (ScyTek Lab, SRS-IFU) were performed on serial sections to assess histological features and to show collagen specificity. Picro-Sirius Red was also analyzed under polarized light using Olympus Upright BX63 microscope. Picro-Sirius Red quantification was performed using QuPath software (https://qupath.github.io/), by analyzing the percentage of area occupied by Picro-Sirius Red over the entire tumor area.

For colocalization analysis, heat-induced epitope retrieval was performed on deparaffinized sections using a preheated target retrieval solution-High pH for 30 min. Following antigen retrieval, the sections were blocked with 2% normal goat serum in 1X PBS for 60 min at room temperature and then incubated with Collagen 1 (1:100 dilution, Invitrogen #MA1-26771, clone COL-1, RRID AB_2081889) and Gelsolin (1:1500 dilution, Invitrogen, #PA5-29910, RRID AB_2547384) at 4 °C in a humidified chamber. Sections were rinsed in PBS and incubated with corresponding secondary antibodies Alexa Fluor 488 and 647 (1:200, Molecular Probes, Invitrogen Life Technologies) for 1 h at room temperature. To visualize the cell nuclei, slides were counterstained with 4,6-diamidino-2-phenylindole (DAPI, Sigma-Aldrich), mounted with a Phosphate-Buffered Salines/glycerol solution and examined under Olympus Upright BX51 microscope. Colocalization Coefficient was automatically determined by FiJi Software (https://imagej.net/software/fiji/downloads) (Colocalization Index), overlapping the same field with Collagen I and Gelsolin.

For IHC analysis paraffin was removed with xylene and the sections were rehydrated in graded alcohol. Antigen retrieval was carried out using a preheated target retrieval solution for 30 min and endogenous peroxidase activity was quenched with 3% hydrogen peroxide in distilled water for 10 min at RT. Tissue sections were blocked with FBS serum in PBS for 60 min and incubated overnight with primary antibodies (Supplemental Table S11). The antibody binding was detected using a polymer detection kit (GAR-HRP, Microtech) followed by a diaminobenzidine chromogen reaction (Peroxidase substrate kit, DAB, SK-4100; Vector Lab). All sections were counterstained with Mayer’s hematoxylin and visualized using a bright-field microscope (LEICA. DM750).

For double immunofluorescence, tumor sections were incubated overnight with primary antibodies against N-Cadherin (1:100, BD Bioscience #610920, RRID AB_398236), E-cadherin (1:100, BD Bioscience #610182, RRID AB_397581), CD4, CD8, CD19 and FoxP3 (same antibodies used for IHC see Supplemental Table S11 but diluted 1:100). Sections were rinsed in PBS and incubated with corresponding secondary antibodies Alexa Fluor488 and AlexaFluor594 (1:400, Molecular Probes, Invitrogen Life Technologies, Grand Island, New York) for 1 h at room temperature. To visualize the cell nuclei, mouse specimens were counterstained with 4,6-diamidino-2-phenylindole (DAPI, Sigma-Aldrich), mounted with a Phosphate-Buffered Salines/glycerol solution and examined under Olympus Upright BX51 microscope.

### FACS analysis

At least 100,000 cells per sample were acquired using a 13-color FACSCelesta (BD Bioscience) for immune cell population analyses. Lymphocytes were characterized using state-of-the-art markers. Specifically, cells were gated for size, singlets, and then by positive and negative markers: CD3^+^CD4^+^ and CD3^+^CD8^+^ T cells, CD3^-^CD19^+^ B cells. T cells were further analyzed to investigate the exhaustion status (Tim3^+^PD-1^+^), and B cells for positivity to CD74. Antibodies are listed in Supplemental Table S10.

### Single-cell RNA-seq short reads alignment and quality control

Reads of the newly generated samples (which samples) were aligned with CellRanger v6.1.2 to the mm10 reference genome (refdata-gex-mm10-2020-A). The published samples (see Supplemental Table S1) were re-processed, starting from previous CellRanger output (for a detailed description), please refer to the methods section of original publication [14]. The samples from each individual sequencing experiment were individually subject to quality control. Thresholds applied to the different experiments are summarized in Supplemental Table S1. Samples from the CD45-EpCAM- (stromal TME) compartment were decontaminated from ambient mRNAs using SoupX (v1.6.2) [47].

### Single-cell RNA-seq profiles normalization, pre-clustering, and re-assignment

As shown in the analysis scheme **(**Supplemental Fig. S2A), samples from individual experiments were combined for further processing using the Seurat R framework (Seurat v4.3.0) [48]. Normalization was performed on the combined data, using SCTransform (using the 3’000 most variable genes) [49]. Principal Component Analysis (PCA) and Uniform Manifold Approximation and Projection (UMAP), using the first 50 principal components, were performed to reduce dimensionality and for visualization. A SNN graph was constructed based on the first 50 principal components. An initial clustering of cells was performed using the Leiden algorithm [20] (resolution = 0.8). Cells were then reassigned to compartments, based on the assignment to clusters, namely each cluster was associated with the compartment from which most of its cells originated from, and all the cells from the cluster were then assigned to this compartment. Therefore, cells that might have been wrongly associated with a compartment in the sorting process, would be re-assigned based on their gene expression profiles.

### Single-cell RNA-seq data clustering

Separately for each compartment, PCA was performed and a SNN graph was constructed based on the first 50 principal components. Cells were clustered at different resolutions (between 0.2 and 2, with steps of 0.1) with the Leiden algorithm [20] and the individual partitions were scored using the silhouette score (clusterCrit R package v1.2.8) and the first 60 principal components (as previously described in [21]). Based on the silhouette scores and the number of identified clusters for each partition, the optimal resolutions were defined as 1, 0.4 and 0.5 for CD45^+^EpCAM^-^ (immune TME), CD45^-^EpCAM^+^ (cancer cells) and CD45-EpCAM- (stromal TME) compartments, respectively. Cluster stability was further confirmed by an orthogonal method, clustree (v0.5.0) [22].

### Single-cell RNA-seq marker gene identification and cell type annotation

Marker genes (positive and negative) for each cluster were identified using the FindAllMarkers() function from the Seurat package with min.pct = 0.1 and logfc.threshold = 0.1 (Supplemental Table S4) using a Wilcoxon test. Cell type annotation was guided by automatically assigning cell types to clusters using scType [23] with an extended marker gene database (Supplemental Table S3). These labels were manually curated by comparing computationally identified marker genes to manually curated sets of marker genes.

### Single-cell RNA-seq dimensionality reduction and visualization

UMAP was applied (using the Seurat implementation RunUMAP() with default parameters) on the first 50 principal components of each compartment, and used solely for visualization purposes.

### Tabula Muris Senis data re-analysis

Re-analysis of the *Tabula Muris Senis* (TMS) data [31] was performed on the pre-filtered count matrix from the mammary gland data (droplet-based) using a similar approach as the one outlined above. In a nutshell, normalization was performed using SCTransform [49], optimal clustering resolutions were determined using a combination of different approaches: silhouette score and clustree [22], and additionally a re-implementation of multiK [50]. Two optimal numbers of clusters were picked (20 and 29 at resolutions of 0.3 and 1.1, respectively) for downstream analysis. Clustering was performed using the Leiden algorithm [20]. For downstream analyses the immune cells were split from all other cell types, based on the cell-type label of the majority of cells in each of the identified clusters (cell-type labels as provided in the original publication of the TMS dataset). Marker gene identification and differential gene expression was performed as described above. Gene set enrichment analysis using the age-dependent CAF signature identified in this study was performed using the enricher function from the clusterProfiler package, v4.4.4 [51].

### Cell-cell communication analysis

Cell-cell communication was predicted using the LIANA [34] framework (v0.1.13). First, conversion from mouse gene symbols to the corresponding human orthologs was performed based on the OmniPath resource [52], following the guidelines provided in the LIANA tutorials. After that, the LIANA workflow was applied on each age group separately at the level of sub-compartments. All tumor cells were combined into one sub-compartment to increase cell numbers. Only sub-compartments with more than 10 cells were used for the analysis. For comparing the results of young and adult samples, we computed an interaction score defined as the product of the interaction specificity (natmi edge specificity) and expression magnitude (sca.LRscore). We further computed the Jaccard index (similarity between the identified interactions for young and adult cell population pairs) with increasing thresholds on the aggregated rank (as reported by LIANA) of predicted interactions (0.05 to 1 with steps of 0.05).

### Proportion tests

Deviations from expected proportions of cells from different age/treatment groups in each cluster were statistically identified (scProportionTest v0.0.0.9000, [25]). In a nutshell, this method uses a permutation test to calculate a *p*-value for the deviation of expected proportions of cells from different samples in each cluster and provides a confidence interval using bootstrapping. The method was applied with default parameters and 1’000 permutations.

### Aged-CAF signature derivation

The signature was derived by overlapping the differentially expressed genes (logfc.threshold = 0.25; adjusted *p*-value < 0.05) obtained from (1) comparing matrix CAFs cluster 2 (enriched for adult cells) and 6 (enriched for young cells) and (2) comparing all adult to all young CAFs, after excluding clusters that, despite showing a CAF signature, displayed small, residual levels of contaminants (clusters 1 and 5).

### Gene set enrichment analyses

Analyses were run using the R package clusterProfiler (v3.18.1) [51] and annotation from enrichR (v3.2) [53]. Analyses were run using either KEGG [28], the Hallmark gene sets [29], Reactome [54], and/or ChEA regulons [32], as reference annotation(s).

### Published scRNA-seq mining

For the data from Pal et al. [44], the Seurat object SeuratObject_TNBCSub.rds was downloaded from figshare (https://figshare.com/s/c584dda937d346cc9a80). The signatures of interest were scored at the single-cell level using the AddModuleScore() function from Seurat (default parameters, except for ctrl = 10). Cell type annotation was transferred from the metadata reported in the original publication. For the data from Wu et al. [45] the original count matrix was downloaded from the GEO, and the metadata were extracted from the supplementary information of the original publication. Based on this metadata, a standard, basic Seurat pipeline (consisting of NormalizeData(), ScaleData(), and FindVariableFeatures()) was re-run on the quality-filtered, non-cancerous cells, reported in the paper, using defaults parameters. Single CAFs were scored for the signatures of interest, in the same way described above for Pal et al. For both studies, to stratify the single-cell profiles in adult and young donors, a cutoff on age = 50 was considered.

Random forest classifiers to distinguish individual CAFs from adult and young donors, were trained using the R package tidymodels (v1.1.1). Training and testing was performed using the normalized expression of the genes in the aged-CAFs signature as features. The input sets were balanced (Wu et al. 1’000 randomly picked CAFs per group, Pal et al. 400 randomly picked CAFs per group), and split into 50/50 training/test sets. Scaling and centering of the feature values was performed on the training set, and then applied to the test set. Classifiers were trained using the ranger R package (v0.13.1) with importance set to “permutation”. The function vi from the vip package (v0.3.2) was used to extract feature importance (as the average change in performance after random permutations). Importance of the features in the two models (one obtained from Wu et al. data and one from Pal et al. data) were compared at increasingly stringent thresholds of importance. For each threshold, the overlap was computed, and enrichment was measured as the *p*-value of a Fisher’s exact test (using the fisher.test() function from base R).

### Statistical analysis

Data were expressed as means ± SEM (in case of normal distribution). Normality was assessed using Shapiro Wilk’s normality test. The range of the variance of the distributions being compared was checked, using a Breusch–Pagan test when appropriate. To compare two sample groups, either the Student’s *t*-test or the Mann–Whitney *U*-test was used depending on normality. Statistical analysis was carried out Prism 10 (GraphPad).

## Supplementary information

Supplemental Figures and Legends to Figures and Tables

Suppl. Tab 1

Suppl. Tab 2

Suppl. Tab 3

Suppl. Tab 4

Suppl. Tab 5

Suppl. Tab 6

Suppl. Tab 7

Suppl. Tab 8

Suppl. Tab 9

Suppl. Tab 10

Suppl. Tab 11

Suppl. Tab 12

## Data Availability

scRNA-seq datasets have been deposited in the NCBI GEO database [55] under the identifier GSE252968. Objects with processed data tables are available on Zenodo (https://zenodo.org/records/13960874).

## Code availability

The scripts to reproduce the analyses shown in this manuscript are available on the following Github repository: https://github.com/barozziLab/AgingBreastTME.
