# Supplementary material for: Age-dependent differences in breast tumor microenvironment: challenges and opportunities for efficacy studies in preclinical models
Source: Cell Death Differ. 2025 Jan 27;32(6):1000–13. doi: 10.1038/s41418-025-01447-1 (PMC12162869; doi:10.1038/s41418-025-01447-1)
Supplement: Supplementary file 1 — Supplemental Figures and Legends to Figures and Tables [file 41418_2025_1447_MOESM1_ESM.pdf]

Supplemental Materials

Supplemental Figures and Legends

Figure S1

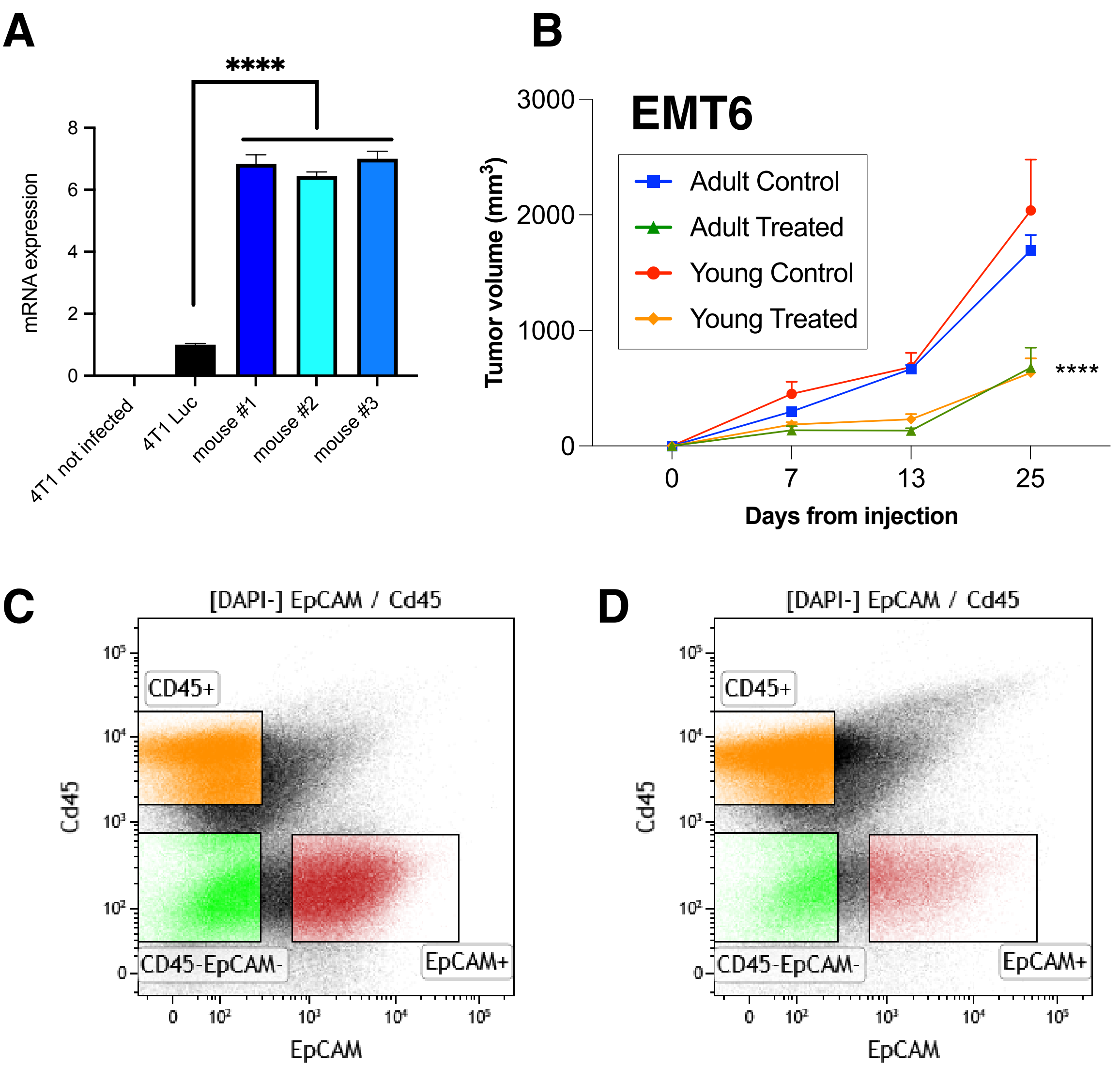

Gate	%Total	%Gated
All	22,9	100,0
CD45-EpCAM-	3,6	15,6
CD45+	8,0	34,9
EpCAM+	4,5	19,5

Gate	%Total	%Gated
All	36,7	100,0
CD45-EpCAM-	3,4	9,2
CD45+	19,3	52,6
EpCAM+	2,4	6,6

Figure S1. (A) Quantification of luciferase levels in 4T1 tumors. Left: Quantification of luciferase mRNA by qPCR in three different 4T1-Luc tumors (blue bars) from 3 mice, the 4T1-Luc cell line (black bar) as a positive control, and the 4T1 WT cell line as a negative control.

Reactions were performed in triplicate, in three different tumors. A Student's *t*-test was applied, *p*-value < 0.0001 (****) **(B)** Comparison of tumor growth in untreated and TT-treated young vs adult mice (EMT6 model; n= 5; ****: *p*-value < 0.0001; Student's *t*-test). **(C-D)** FACS plot of representative young (B) and adult (C) control mouse in 4T1 model. On the y-axis cells are sorted for the positivity towards CD45, while on the x to the one towards EpCAM. Immune cells are gated as $CD45^{+}EpCAM^{-}$, tumor cells as $CD45^{-}EpCAM^{+}$, and stromal cells as double negative population.

Figure S2

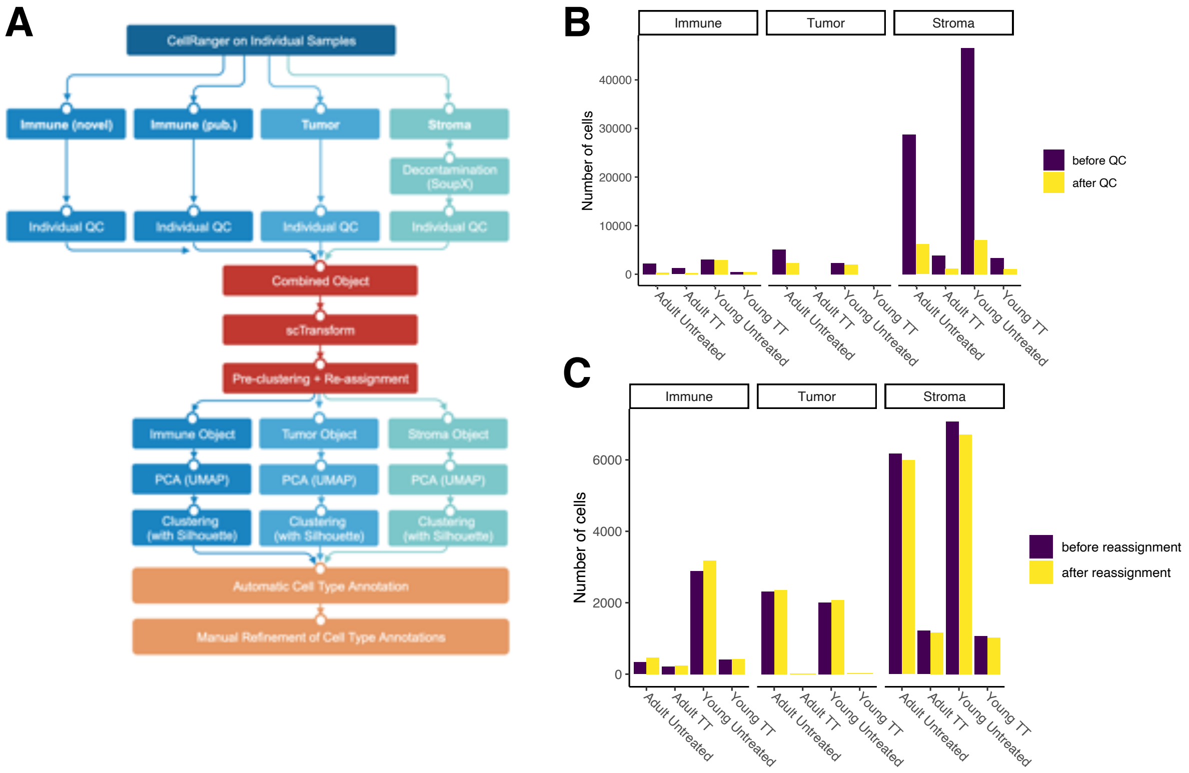

Figure S2. (A) Schematic of the basic computational pipeline employed to process, consolidate, and annotate the scRNA-seq profiles. **(B)** Bar plots showing the number of cells, per compartment (immune TME, tumor, stroma), per condition (adult/young, TT/untreated), before and after quality controls (QC). **(C)** Same as (B), before and after computational pre-clustering and re-assignment of cells to compartments.

Figure S3

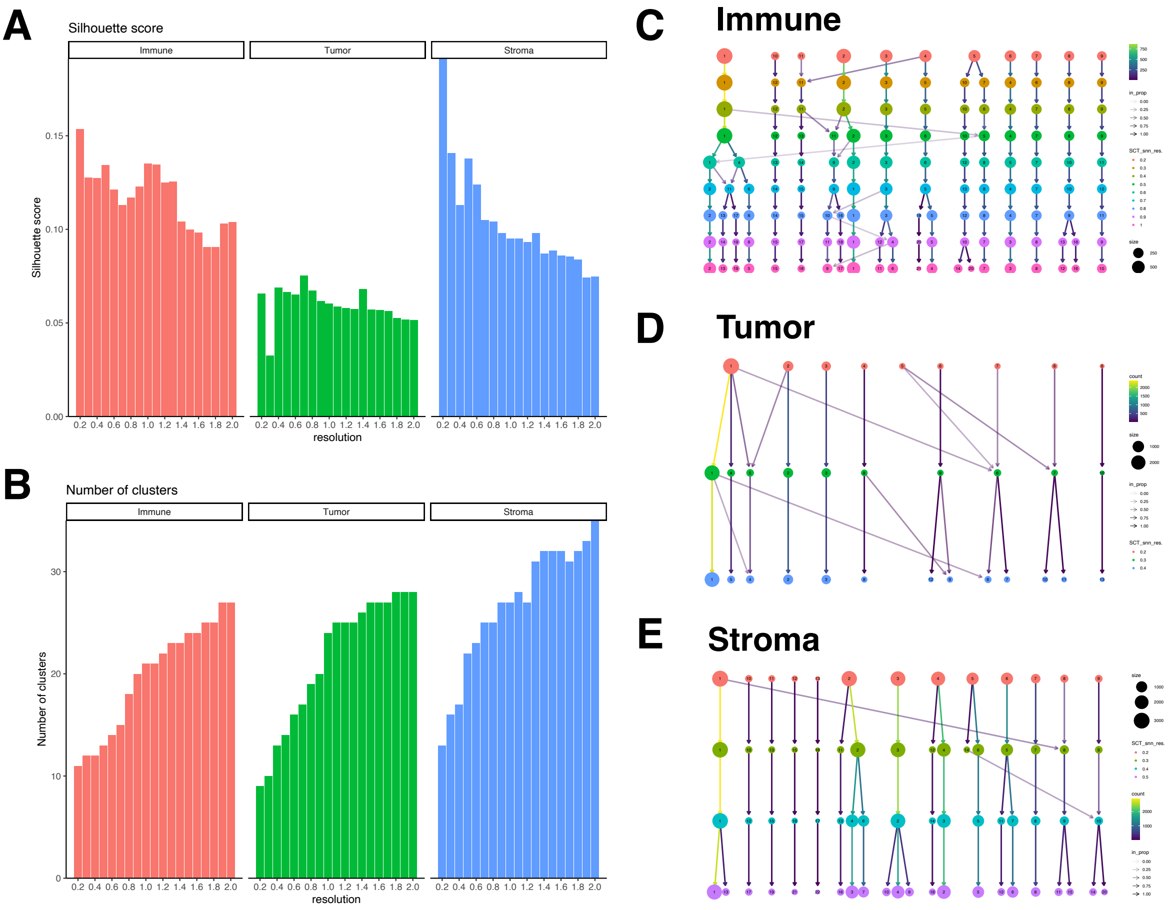

Figure S3. (A) Bar plots showing the silhouette scores obtained at increasing resolution of cell clustering. **(B)** Same as (A) indicating the number of clusters at increasing clustering resolutions. **(C)** Clustree for the immune cells compartment, spanning the range of resolution around the optimal one. **(D)** Same as (C), for tumor cells. **(E)** Same as (C), for stromal cells.

Figure S4

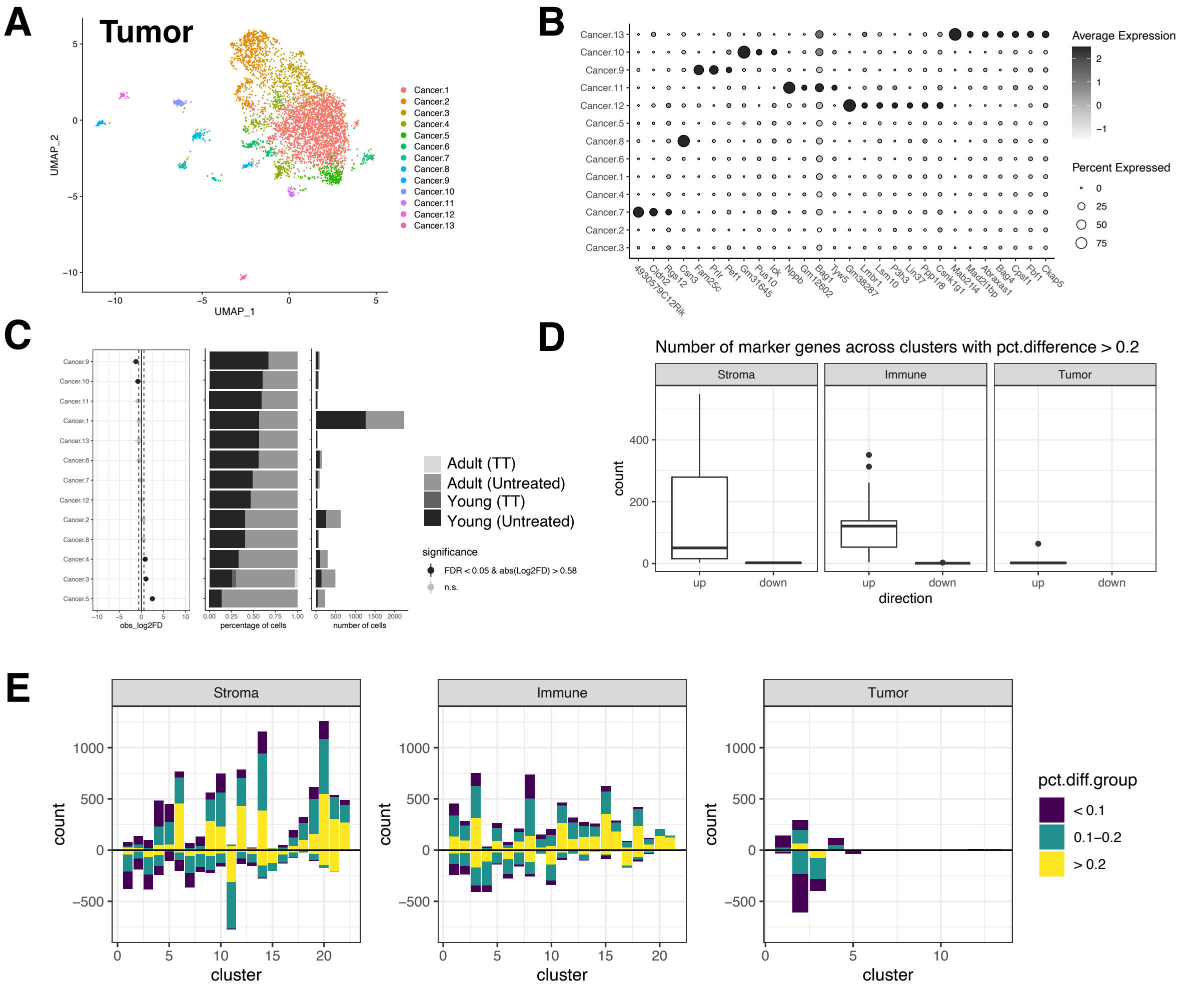

Figure S4. (A) UMAP projection of the filtered tumor cells, color-coded by cluster. **(B)** Dot plots highlighting the marker genes identified for the tumor cells clusters. Size of the dot proportional to the fraction of cells in the indicated cluster showing non-zero expression for the indicated gene. Color shade of the dot shows instead the average normalized expression of the gene in the indicated cluster. **(C)** (left) Relative differences in cell proportions for each tumor cell cluster between cells from adult vs young recipients. Clusters showing a significant difference (FDR < 0.05 and mean | Log2 fold enrichment | > 0.58) are highlighted (permutation test; n=10,000). (right) Stacked bar chart showing the underlying cell composition of each cluster. **(D)** Box plots showing the distributions of number of markers genes (significantly up or down regulated, see **Methods**) identified in each compartment (immune TME, tumor, stroma), showing a significant difference (see **Methods**) and a difference in the number of

cells expressing the gene of at least 0.2 (range 0-1). **(E)** Stack bar charts showing the same information as (D) but including all genes showing a statistically significant difference (stratified by the difference in the number of cells expressing the gene) and separately for each cluster.

Figure S5

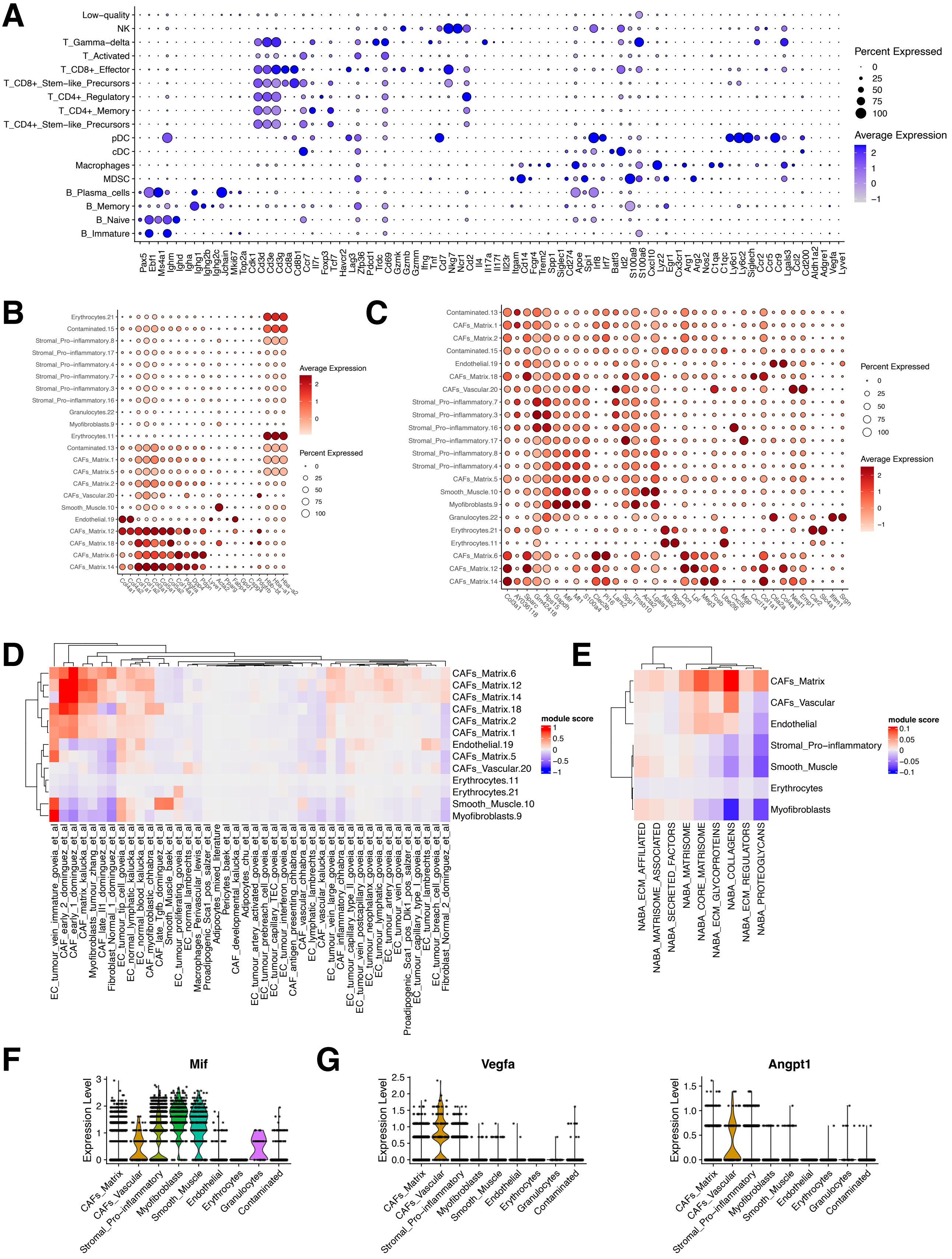

A
Low−quality
NK
T_Gamma−delta
T_Activated
T_CD8+_Effector
T_CD8+_Stem−like_Precursors
T_CD4+_Regulatory
T_CD4+_Memory
T_CD4+_Stem−like_Precursors
pDC
cDC
Macrophages
MDSC
B_Plasma_cells
B_Memory
B_Naive
B_Immature
Pax5 Ebf1 Ms4a1 Ighm Ighd Igha Ighg1 Ighg2b Ighg2c Jchain Mki67 Top2a Cdk1 Cd3d Cd3e Cd3g Cd8a Cd8b1 Ccr7 Il7r Foxp3 Tcf7 Havcr2 Lag3 Zfp36 Pdcd1 Trdc Cd69 Gzmk Gzmb Gzmm Ifng Tnf Cd7 Nkg7 Ncr1 Cd2 Il4 Il17a Il17f Il23r Itgam Cd14 Fcgr4 Trem2 Spp1 Siglec1 Cd274 Apoe Spi1 Irf8 Irf7 Batf3 Id2 S100a9 S100a6 Cxcl10 Lyz2 Egr1 Cx3cr1 Arg1 Arg2 Nos2 C1qa C1qc Ly6c1 Ly6c2 Siglech Ccr2 Ccr5 Ccr9 Lgals3 Cd2 Cd200 Aldh1a2 Adgre1 Vegfa Lyve1
Percent Expressed
0
25
50
75
100
Average Expression
2
1
0
−1
B
Erythrocytes.21
Contaminated.15
Stromal_Pro−inflammatory.8
Stromal_Pro−inflammatory.17
Stromal_Pro−inflammatory.4
Stromal_Pro−inflammatory.7
Stromal_Pro−inflammatory.3
Stromal_Pro−inflammatory.16
Granulocytes.22
Myofibroblasts.9
Erythrocytes.11
Contaminated.13
CAFs_Matrix.1
CAFs_Matrix.5
CAFs_Matrix.2
CAFs_Vascular.20
Smooth_Muscle.10
Endothelial.19
CAFs_Matrix.12
CAFs_Matrix.18
CAFs_Matrix.6
CAFs_Matrix.14
Average Expression
2
1
0
Percent Expressed
0
25
50
75
100
C
Contaminated.13
CAFs_Matrix.1
CAFs_Matrix.2
Contaminated.15
Endothelial.19
CAFs_Matrix.18
CAFs_Vascular.20
Stromal_Pro−inflammatory.7
Stromal_Pro−inflammatory.3
Stromal_Pro−inflammatory.16
Stromal_Pro−inflammatory.17
Stromal_Pro−inflammatory.8
Stromal_Pro−inflammatory.4
CAFs_Matrix.5
Smooth_Muscle.10
Myofibroblasts.9
Granulocytes.22
Erythrocytes.21
Erythrocytes.11
CAFs_Matrix.6
CAFs_Matrix.12
CAFs_Matrix.14
Percent Expressed
0
25
50
75
100
Average Expression
2
1
0
−1
D
CAFs_Matrix.6
CAFs_Matrix.12
CAFs_Matrix.14
CAFs_Matrix.18
CAFs_Matrix.2
CAFs_Matrix.1
Endothelial.19
CAFs_Matrix.5
CAFs_Vascular.20
Erythrocytes.11
Erythrocytes.21
Smooth_Muscle.10
Myofibroblasts.9
module score
1
0.5
0
−0.5
−1
E
CAFs_Matrix
CAFs_Vascular
Endothelial
Stromal_Pro−inflammatory
Smooth_Muscle
Erythrocytes
Myofibroblasts
module score
0.1
0.05
0
−0.05
−0.1
NABA_ECM_AFFILIATED
NABA_MATRISOME_ASSOCIATED
NABA_SECRETED_FACTORS
NABA_MATRISOME
NABA_CORE_MATRISOME
NABA_ECM_GLYCOPROTEINS
NABA_COLLAGENS
NABA_ECM_REGULATORS
NABA_PROTEOGLYCANS
F
Mif
G
Vegfa
Angpt1
Expression Level
CAFs_Matrix
CAFs_Vascular
Stromal_Pro−inflammatory
Myofibroblasts
Smooth_Muscle
Endothelial
Erythrocytes
Granulocytes
Contaminated

Figure S5. (A) Dot plot showing the marker genes used to manually annotate the immune cell clusters. Size of the dot proportional to the fraction of cells in the indicated cluster showing non-zero expression for the indicated gene. Color shade of the dot shows instead the average normalized expression of the gene in the indicated cluster. **(B)** Dot plot showing the top marker genes of the identified stromal cell clusters. **(C)** Same as (B) considering the merge of all top 2 markers per cluster. **(D)** Heat map showing the gene expression score (aggregated for all cells in each cluster) for the signatures highlighted on the columns, for each of the one of the indicated clusters. Rows and columns are hierarchically clustered (complete linkage, Pearson's distance). **(E)** Same as (D), using the Naba et al. gene sets (MSigDB). **(F)** Violin plots showing the expression of *Mif* in single cells, for the indicated clusters. **(G)** Same as (F), for *Vegfa* and *Angpt1*.

Figure S6

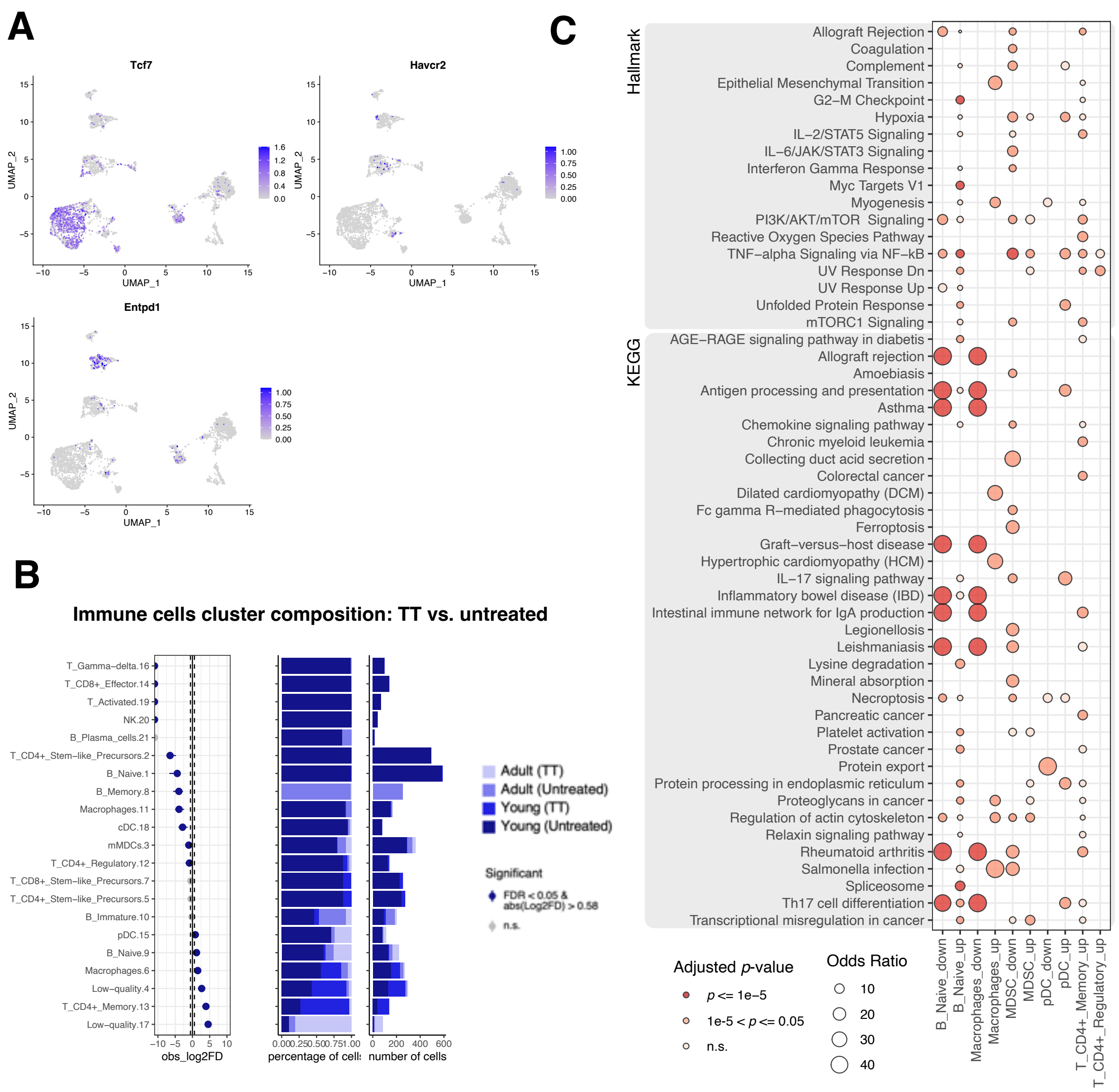

Figure S6. (A) UMAP projection highlighting the gene expression of *Tcf1* (Tcf7) and canonical exhaustion markers (*Havcr2* and *Entpd1*). **(B)** (left) Relative differences in cell proportions for each immune TME cluster between cells from TT-treated vs untreated recipients. Clusters showing a significant difference (FDR < 0.05 and mean | Log2 fold enrichment | > 0.58) are highlighted (permutation test; n=10,000). (right) Stacked bar chart showing the underlying cell composition of each cluster. **(C)** Bubble plot summarizing the results of functional enrichment analyses (using the hallmark gene sets and the KEGG pathways) across the regulated genes (up- and down- regulated separately) in adult vs young mice, per cell type (columns). Size of

the bubble proportional to the odds ratio; color-coded based on statistical significance of the enrichment (adjusted *p*-value; hypergeometric test).

Figure S7

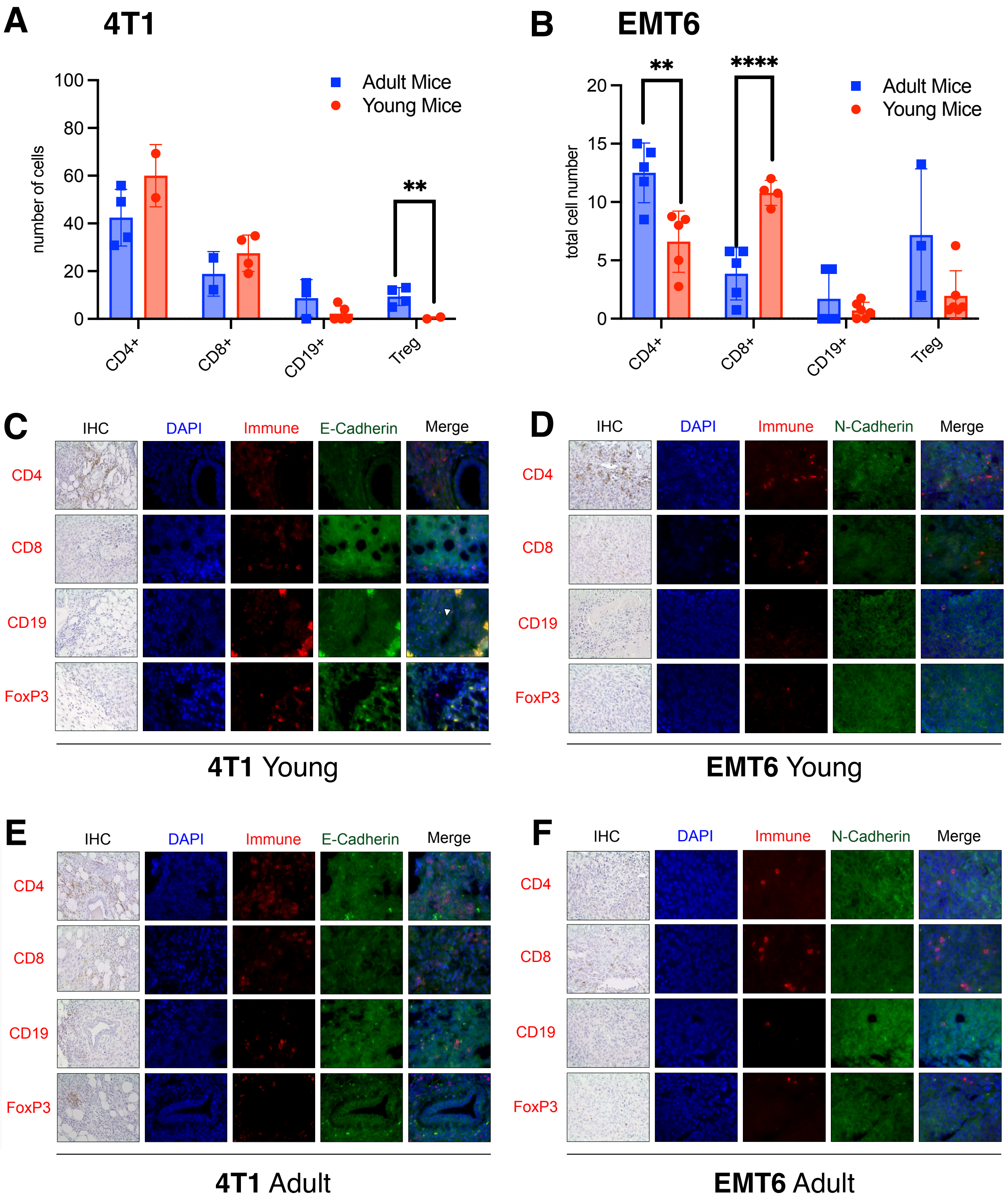

Figure S7. (A) IHC quantification of T helper (CD4$^+$), T cytotoxic (CD8$^+$), B cells (CD19$^+$) and regulatory T cells (Foxp3$^+$). Images were taken in 5 slides per experimental group (young vs adult mice), and for each slide 4 different random fields were counted in the model. (**B)** IHC quantification of T helper (CD4$^+$), T cytotoxic (CD8$^+$), B cells (CD19$^+$) and regulatory T cells

(Foxp3^{+}). Images were taken in five slides per experimental group (young vs adult mice), and for each slide, four different random fields were counted in the EMT6 model. (n= minimum 3 mice per each experimental group. Student *t*-test was applied to test for differences between Young and Adult mice. **p<0.01, ****p<0.0001. **(C)** On Top panel shows IF and IHC are shown in young mice for 4T1 tumor. CD4 T helper cells, CD8 Cytotoxic T cells, CD19 B cells and T reg FoxP3 are highlighted by the respective markers shown. For each staining DAPI is shown in blue, immune cells in red, tumor cells marked with E-Cadherin in green, followed by the merge of the three stainings. (**D)** On Top panel shows IF and IHC are shown in young mice for EMT6 tumor. CD4 T helper cells, CD8 Cytotoxic T cells, CD19 B cells and T reg FoxP3 are highlighted by the respective markers shown. For each staining DAPI is shown in blue, immune cells in red, tumor cells marked with N-Cadherin in green, followed by the merge of the three stainings. (**E)** On Top panel shows IF and IHC are shown in adult mice for 4T1 tumor. CD4 T helper cells, CD8 Cytotoxic T cells, CD19 B cells and T reg FoxP3 are highlighted by the respective markers shown. For each staining DAPI is shown in blue, immune cells in red, tumor cells marked with E-Cadherin in green, followed by the merge of the three stainings. (**F)** On Top panel shows IF and IHC are shown in adult mice for EMT6 tumor. CD4 T helper cells, CD8 Cytotoxic T cells, CD19 B cells and T reg FoxP3 are highlighted by the respective markers shown. For each staining DAPI is shown in blue, immune cells in red, tumor cells marked with N-Cadherin in green, followed by the merge of the three stainings.

Figure S8

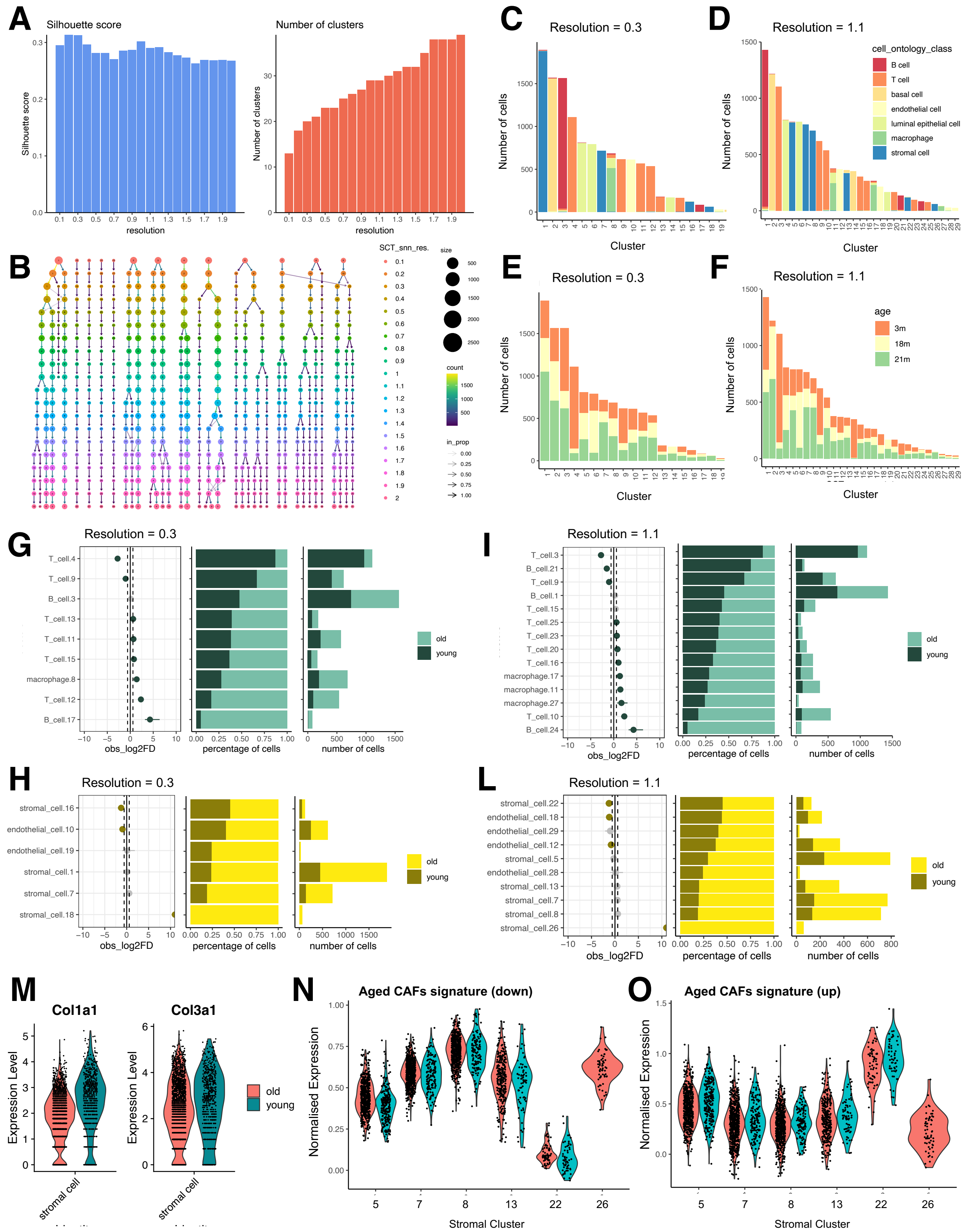

Figure S8. (A) (left) Bar plots showing the silhouette scores obtained at increasing resolution of cell clustering of the TMS data from normal mammary glands, of mice of different ages.

(right) Same as (A) indicating the number of clusters obtained at increasing clustering resolutions. **(B)** Clustree spanning the range of resolutions around the two optimal ones (0.3, 1.1). **(C)** Bar plots showing the total number of cells per cluster, split by cell-type annotation, for resolution 0.3. **(D)** Same as (C), for resolution 1.1. **(E)** Bar plots showing the total number of cells per cluster, split by age of the mouse, for resolution 0.3. **(F)** Same as (E), for resolution 1.1. **(G)** (left) For resolution 0.3, elative differences in cell proportions for each immune TME cluster between cells from the mammary gland of old (18 or 21 months old) and young (3 months old) mice. Clusters showing a significant difference (FDR < 0.05 and mean | Log2 fold enrichment | > 0.58) are highlighted (permutation test; n=10,000). (right) Stacked bar chart showing the underlying cell composition (relative or absolute) of each cluster. **(H)** Same as (G) but for stromal cells, including endothelial. **(I)** Same as (G), but considering the clusters obtained at resolution 1.1. **(L)** Same as (H) for resolution 1.1. **(M)** Violin plots showing the expression of *Col1a1 and Col3a1* in single cells, for the stromal cells of the normal mammary gland, stratified by age of the mouse (old: 18 or 21 months old; young: 3 months old). **(N)** Violin plots showing the aggregated, normalized expression of the down-regulated genes in the aged CAFs signature identified in this study, across the stromal clusters (excluding endothelial and epithelial cells) identified at clustering resolution 1.1, and stratified by age of the mouse. **(O)** Same as (N) but considering the up-regulated genes in the aged CAFs signature identified in this study.

Figure S9

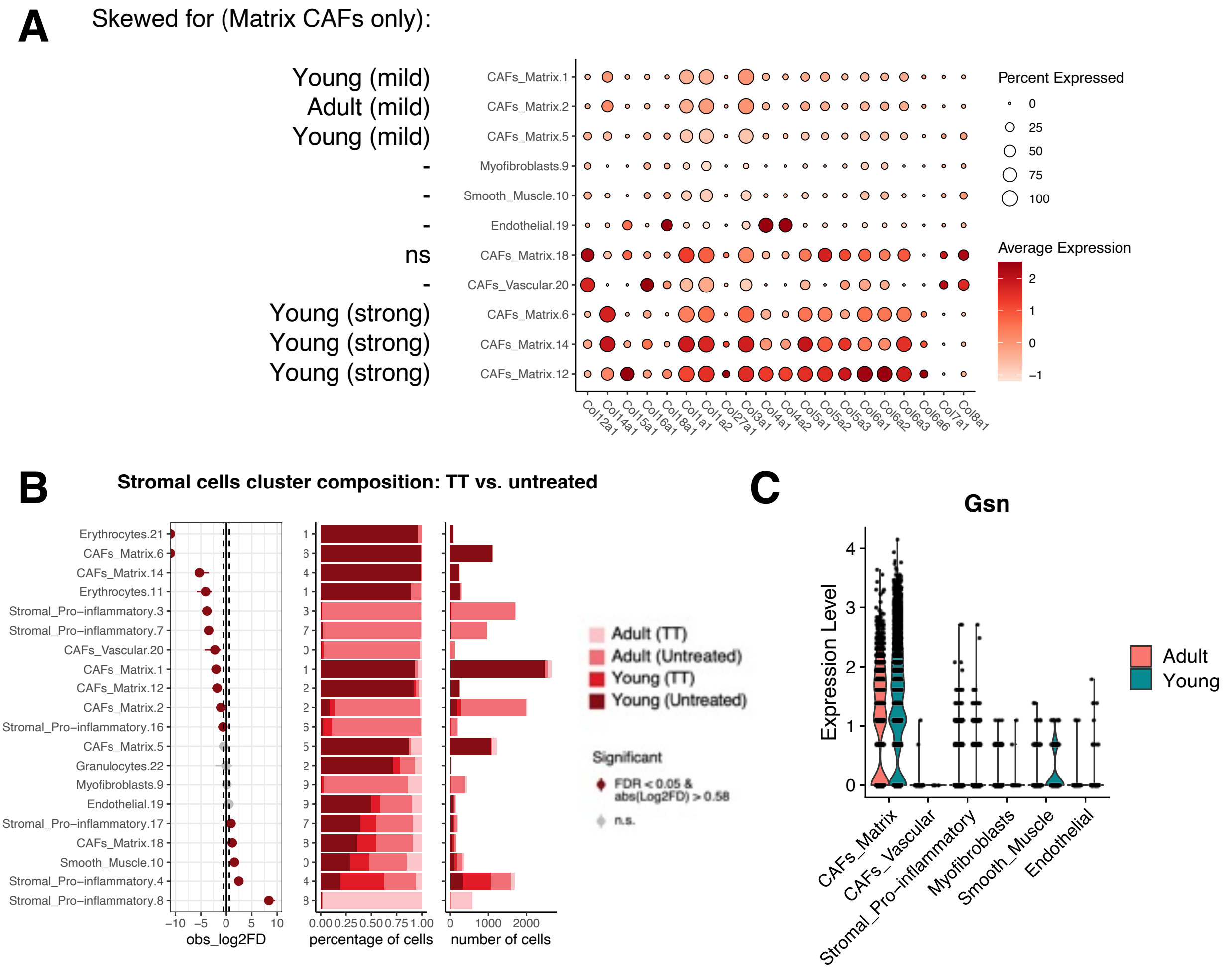

Figure S9. (A) Dot plot showing the pattern of expression of collagen genes across the clusters identified in the stroma. Size of the dot proportional to the fraction of cells in the indicated cluster showing non-zero expression for the indicated gene. Color shade of the dot shows instead the average normalized expression of the gene in the indicated cluster. **(B)** (left) Relative differences in cell proportions for each stromal cell cluster between cells from TT-treated vs untreated recipients. Clusters showing a significant difference (FDR < 0.05 and mean | Log2 fold enrichment | > 0.58) are highlighted (permutation test; n=10,000). (right) Stacked bar chart showing the underlying cell composition of each cluster. **(C)** Violin plots showing the expression of *Gsn* (gelsolin) in single cells, for the indicated clusters (split for adult vs young).

Figure S10

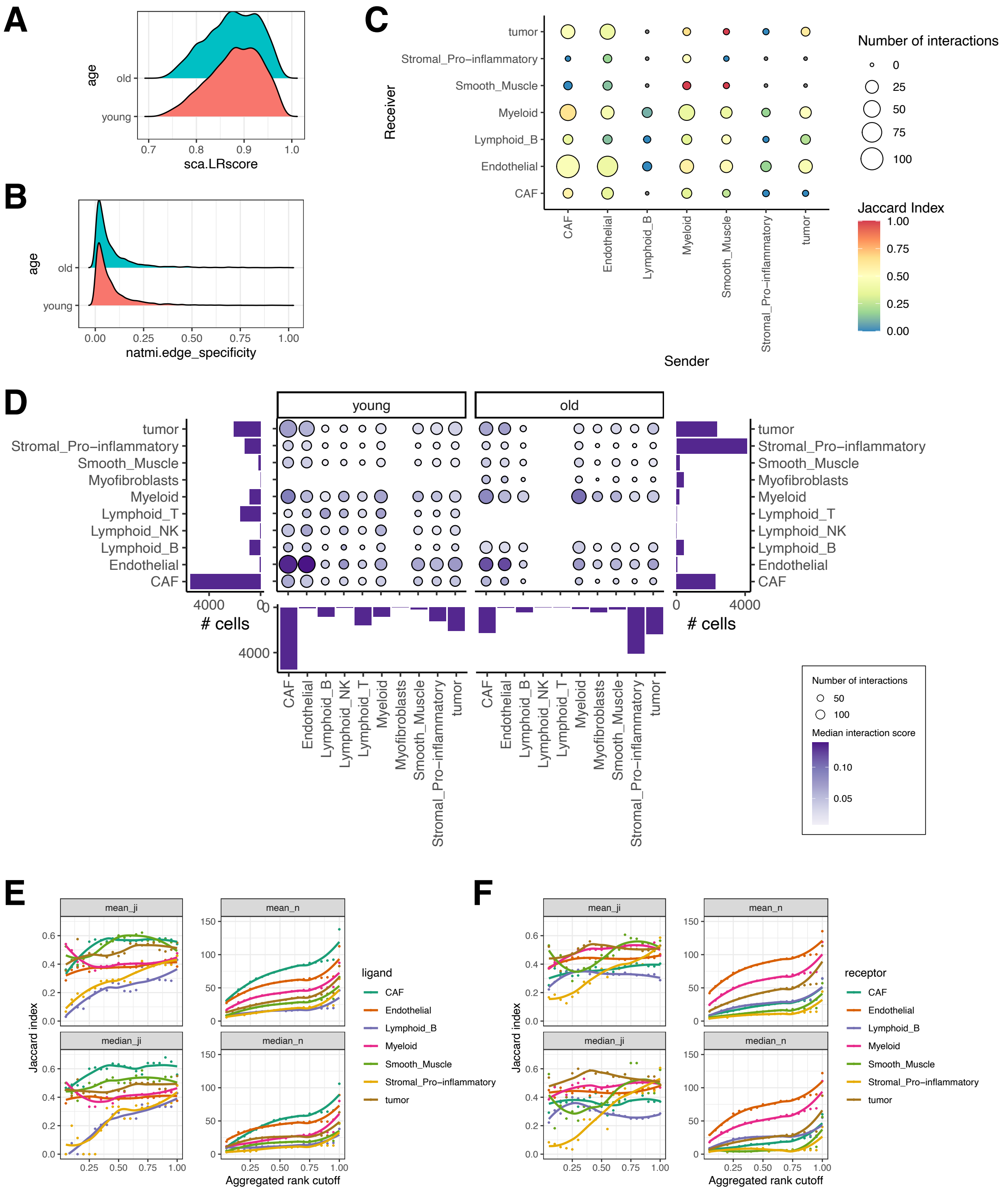

Figure S10. (A) Distributions of expression magnitude (sca.LRscore) for the interactions predicted in the TME of young (red) and adult (green) mice. **(B)** Distributions of interaction specificity (natmi.edge_specificity) for the interactions predicted in the TME of young (red) and

adult (green) mice. **(C)** Bubble plot summarizing the similarity across pairs of cells populations grouped by the indicated sub-compartments, between predicted interactions in young vs. adult mice. Bubble size indicating the number of interactions, color indicating the similarity as measured by the Jaccard index. **(D)** Bubble plot summarizing the median interaction scores (specificity * sensitivity, color of the bubbles) and numbers of predicted interactions (size of the bubbles) across pairs of cells populations grouped by the indicated sub-compartments, separately in young and adult mice. Bar charts indicated cell numbers per group. **(E-F)** Mean (upper left panels) and median (lower left panels) similarity (Jaccard index) across all sub-compartment pairs with the respective ligand (E) or receptor (F) population, considering the similarity between predicted interactions for young and adult mice. Mean (upper right panels) and median (lower right panels) numbers of interactions across all pairs with one ligand (E) or receptor (F). Individual data points and a smoothing curve fitted by loess regression are shown.

Figure S11

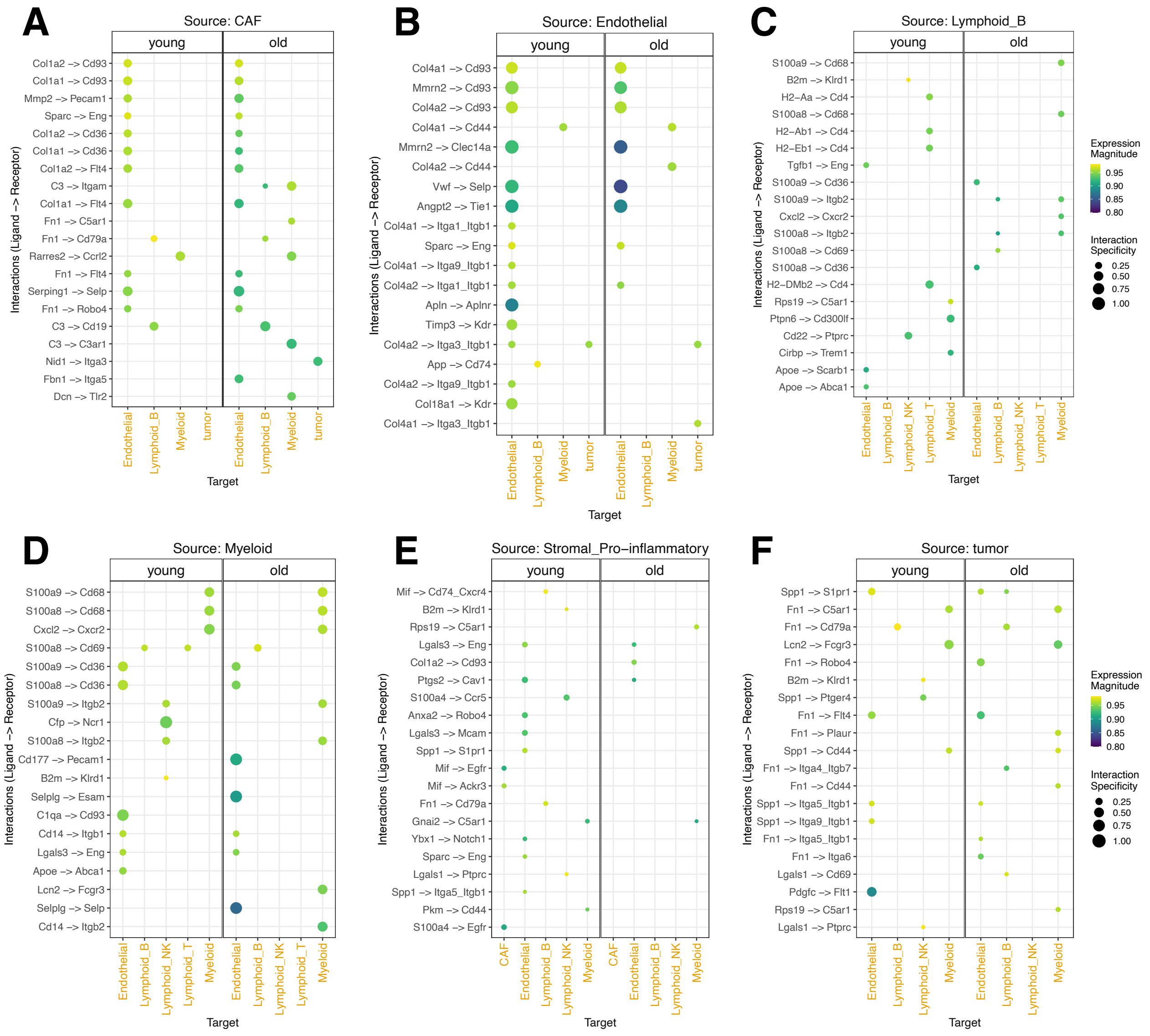

Figure S11. (A-F) Bubble plots comparing the top 20 predicted interactions of the respective source populations in young and adult mice. Size of the bubbles indicates interaction specificity (natmi.edge_specificity) and color indicates expression magnitude (sca.LRscore).

Figure S12

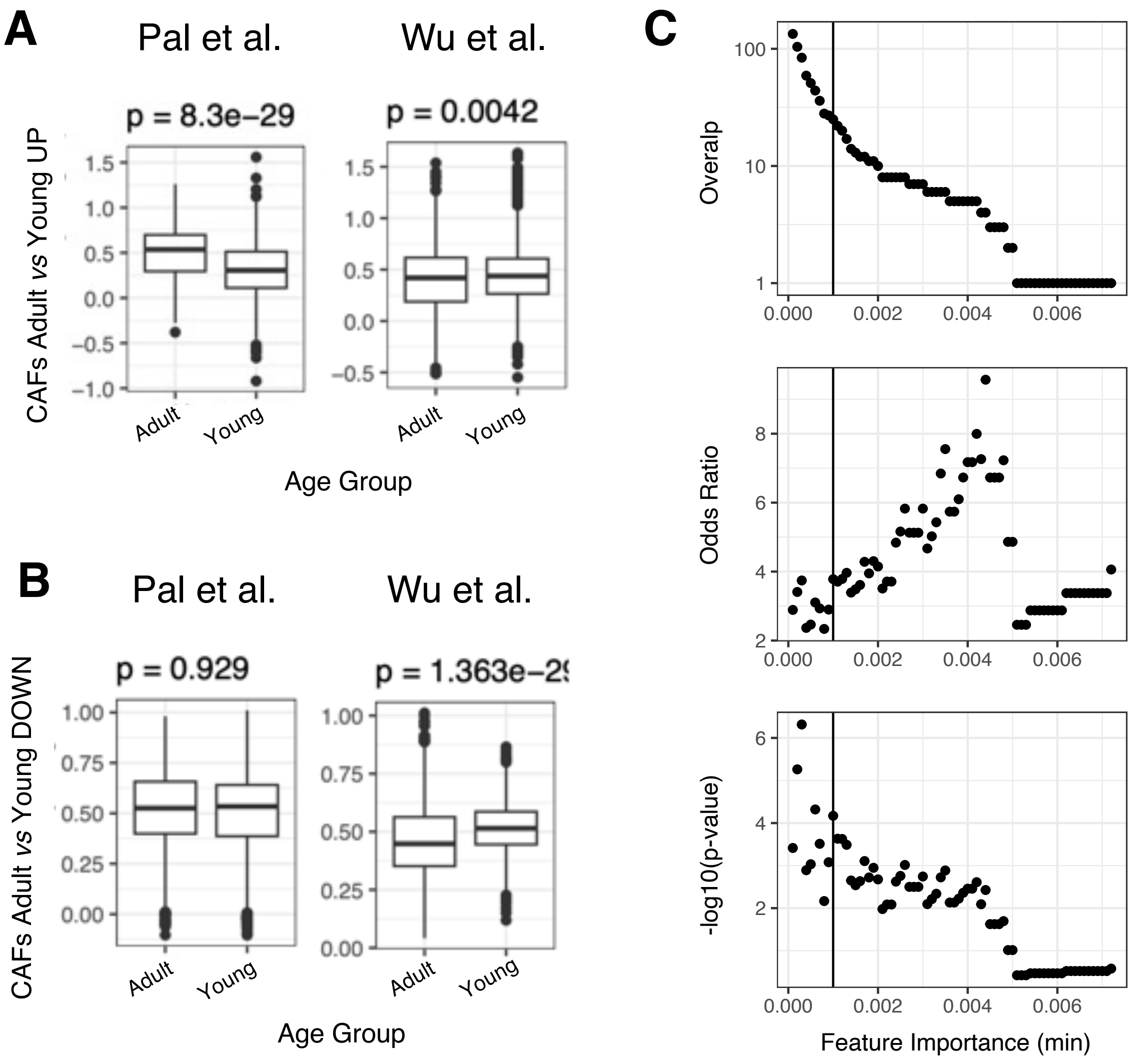

Figure S12. (A) Box plots showing the expression of the up-regulated axis of the adult vs young CAFs signature, in human CAFs from published scRNA-seq data from Pal et al. and Wu et al (*p*-value indicated on top of each plot; Wilcoxon test). **(B)** Same as (A) but considering the down-regulated axis of the adult vs young CAFs signature. **(C)** Scatterplots summarizing the overlap between the most important features in the random forest classifiers trained separately on the Wu et al. and Pal et al. to distinguish CAFs from adult and young TMEs. The plots show (top to bottom) the number of overlapping features between the two models, the odds ratio of this overlap, and its nominal *p*-value (Fisher's exact test), as a function of increasingly stringent threshold applied on the feature importance values.

Supplemental Table Legends

Supplemental Table 1. (S1A) For each single-cell capture, a unique sample identifier is provided, followed by information of the compartment ($EpCAM^+Cd45^-$: tumor; $EpCAM^-Cd45^+$: immune TME; $EpCAM^-Cd45^-$: stroma), age of the recipient mice (young, adult) and whether it was subject to TT, the status (whether the capture was newly generated in this study, or if it were previously published and re-analysed), the overall sequencing depth, followed by the number of captured cells estimated by cellRanger, and the average reads, detected genes, and unique molecular identifiers (UMIs) per cell. After that, Threshold_nCount indicates the lower and upper thresholds applied to the number of UMIs, for a cell to be considered. Similarly, Threshold_nFeature indicates the lower and upper thresholds applied to the number of detected genes, for a cell to be considered. These are followed by information on other thresholds applied at the QC stage, and whether SoupX was applied to the sample. At last, the number of cells, with the corresponding average UMIs and detected genes per cell, before and after QC, are indicated.

Supplemental Table 2. (S2A) For each compartment ($EpCAM^-Cd45^+$: immune TME, $EpCAM^+Cd45^+$: tumor, $EpCAM^-Cd45^-$: stroma) the obtained clusters of single cells are indicated. For each cluster, an arbitrary number (as identifier for the cluster), the sub-compartment (a coarse-grained annotation of the cluster), a more fine-grained annotation (Cell-type_label), and the total number of cells in the cluster, are provided.

Supplemental Table 3. (S3A) Extended scType database used in this study. The format is the same as described in the original scType publication, with cellName and shortName indicating the cell type (divided by tissue, tissueType). geneSymbolmore1 and geneSymbolmore2 list the gene symbols of the positively and negatively associated cell markers, respectively. **(S3B)** Manually curated list of marker genes used for the refinement of the annotation of the clusters of the immune TME. **(S3C)** List of marker genes used for the

refinement of the annotation of the clusters of the stroma. The markers were systematically extracted from the literature, as indicated. For each study (Study_ref, Study_link) one or more signatures are present (Cell_type). For each signature, the gene symbols and the species are indicated, along with the exact source of the genes (Notes; supplementary table, text, figure).

Supplemental Table 4. (S4A) Marker genes for each cluster of the immune TME (EpCAM$^-$Cd45$^+$). For each cluster, significantly up- and down-regulated genes (as compared to the cells in other clusters) are listed. For each gene, the log2-fold-change (log2FC), the fraction of cells in the cluster showing non-zero expression for the gene (pct.1), the fraction of cells in other clusters showing non-zero expression for the gene (pct.2), the difference between these two (pct.difference), the *p*-value (Wilcoxon test) and the multiple-hypotheses corrected *p*-values (pvalue.adj) are indicated. **(S4B)** Same as S4A but considering the cancer cells (EpCAM$^+$). **(S4C)** Same as S4A but considering the cells of the stroma (EpCAM$^-$Cd45$^-$).

Supplemental Table 5. (S5A) Summary of the results of scProportionTest considering the cells in the immune TME (EpCAM$^-$Cd45$^+$), comparing the proportion of cells in the TME of tumors from adult vs young mice, for each cluster. For each cluster, the fraction of cells in adult and young mice are shown, followed by the log2-fold-change (obs_log2FD), *p*-value, FDR, as well as mean and confidence interval of the log2-fold-change estimated by bootstrapping. **(S5B)** Same as S5A but comparing the proportion of cells in the TME of tumors from TT-treated vs untreated mice. **(S5C)** Same as S5A but considering the cells in the stroma (EpCAM$^-$Cd45$^-$). **(S5D)** Same as S5B but considering the cells of the stroma (EpCAM$^-$Cd45$^-$). **(S5E)** Same as S5A but considering the cancer cells (EpCAM$^+$). **(S5F)** Same as S5B but considering the cancer cells (EpCAM$^+$).

Supplemental Table 6. (S6A) Marker genes identified in specific immune TME populations (Cell-Type), comparing cells from adult vs young mice. For each population, gene symbols of up- and down-regulated genes are indicated. **(S6B)** Gene set enrichment analysis considering

the up- and down- regulated set from S6A. For each Ontology and Term, the overlapping fraction of genes (Overlap), the *p*-value (P.value; hypergeometric test), the multiple-hypothesis testing corrected *p*-value (Adjusted.P.value), the corresponding odds ratio (Odds.Ratio), a score combining the odds ratio with the statistical significance, and a list of corresponding human orthologs for the overlapping genes (Genes) are provided.

Supplemental Table 7. (S7A) Clusters identified in the *Tabula Muris Senis* (TMS) single-cell transcriptomics data from mammary gland, at low resolution (0.3). For each cluster, a numeric identifier, the assigned cell-type, and the number of cells, are indicated. **(S7B)** Same as S7A, but for resolution 1.1. **(S7C)** Summary of the results of scProportionTest considering the cells in the immune cell populations (resolution 0.3) and comparing the proportion of cells in the immune compartment from old vs young mammary glands, for each cluster. The fraction of cells in adult and young mice are shown, followed by the log2-fold-change (obs_log2FD), *p*-value, FDR, as well as mean and confidence interval of the log2-fold-change estimated by bootstrapping. **(S7D)** Same as S7C, but the non-immune cell populations. **(S7E)** Same as S7C, but for resolution 1.1. **(S7F)** Same as S7D, but for resolution 1.1. **(S7G)** For each cluster (resolution 0.3), significantly up- and down-regulated genes (as compared to the cells in other clusters of the same compartment) are listed. For each gene, the log2-fold-change (log2FC), the fraction of cells in the cluster showing non-zero expression for the gene (pct.1), the fraction of cells in other clusters showing non-zero expression for the gene (pct.2), the difference between these two (pct.difference), and the multiple-hypotheses corrected *p*-value (pvalue.adj; Wilcoxon test) are indicated. **(S7H)** Same as S7G, but for resolution 1.1. **(S7I)** Differentially expressed genes between cells from old vs young mammary gland, for each one of the indicated clusters (resolution 1.1). The same information provided in S7G-H is provided. **(S7L)** Overlap between the age-related DEGs in the TMS data and the aged-CAF signature defined in this study. Number of overlapping genes, along with the corresponding fraction, and adjusted *p*-values (hypergeometric test) are indicated.

Supplemental Table 8. (S8A) Differentially expressed genes identified by comparing cells in cluster 2 vs cluster 6 of the stroma (EpCAM$^-$Cd45$^-$). For each gene, the log2-fold-change (log2FC), the fraction of cells in the cluster showing non-zero expression for the gene (pct.1), the fraction of cells in other clusters showing non-zero expression for the gene (pct.2), the difference between these two (pct.difference), the *p*-value (Wilcoxon test) and the multiple-hypotheses corrected *p*-values (pvalue.adj) are indicated. **(S8B)** Same as S8A but resulting from the comparison between all cells annotated as matrix CAF in adult vs young mice. **(S8C)** Aged-CAF signature genes. **(S8D)** Gene set enrichment analysis considering the up- and down- regulated set from S8C. For each Ontology and Term, the overlapping fraction of genes (Overlap), the *p*-value (P.value; hypergeometric test), the multiple-hypothesis testing corrected *p*-value (Adjusted.P.value), the corresponding odds ratio (Odds.Ratio), a score combining the odds ratio with the statistical significance, and a list of corresponding human orthologs for the overlapping genes (Genes) are provided. **(S8E)** Enrichment analysis of the up- and down- regulated set from S8C, specifically considering the matrisome annotation from Naba et al. 2012. For each Term, the number of overlapping genes (Count), the corresponding odds ratio (Odds.Ratio), the *p*-value (pvalue; hypergeometric test), the multiple-hypothesis testing corrected *p*-value (pvalue.adjust), and a list of corresponding human orthologs for the overlapping genes (Genes) are provided.

Supplemental Table 9. (S9A) Unfiltered cell-cell communication predictions from LIANA. Separately for each age group (old, young) the interactions (source, target) predicted between cells of the indicated types are listed, along with the method-specific metrics quantifying the strength of each interaction.

Supplemental Table 10. (S10A) Gating strategy and reagents used for FACS-sorting of the cells in the three tumor compartments considered in this study. For each compartment, the immune phenotype and the fluorophores are indicated, along with details on each antibody used (clone, catalog number, RRID, and company producing it). **(S10B)** Gating strategy and

reagents for FACS-sorting of lymphocytes of the TME. For each population, the immune phenotype and the fluorophores are indicated, along with details on each antibody used (clone, catalog number, RRID, and company producing it).

Supplemental Table 11. (S11A) Reagents used for IHF and/or IF experiments. For each marker gene considered, dilution, clone, catalog number, RRID, and company producing it, are indicated. The table also indicates whether a reagent was used in IHC experiments, IF experiments, or both.

Supplemental Table 12. (S12A) Performances of the random forest classifiers trained to distinguish single CAFs in the adult TME from those in the young TME, based on the age-related CAFs signature. The dataset, the metric, and the value for the metric (0-1) are listed. **(S12B)** Summary of the feature importance in the two models. The variable (gene symbol) along with the importance value for the two models (Pal et al. and Wu et al.), whether the feature is identified as one of the most important one in both models (importance >= 0.001), and whether the gene was part of either the up- or the down-regulated genes in age-related CAFs signature, are indicated. **(S12C)** Source data for Suppl. Fig. S12C.
